# Kinetochore proteins control microtubule dynamics in postmitotic neurons to regulate the formation of dendritic spines

**DOI:** 10.1073/pnas.2520684123

**Published:** 2026-04-27

**Authors:** Guoli Zhao, Aditi Sharma, Jing Tang, Martina Aleman, Xing Liang, Lauren Miner, Jingqi Qi, Wangchu Xiang, Feng Tian, Yves Goldberg, Zhigang He, Kang Shen, Leticia Peris, Thomas Schwarz

**Affiliations:** 1. F.M. Kirby Neurobiology Center, Boston Children’s Hospital, Boston, MA 02115, USA; 2. Univ. Grenoble Alpes, Inserm, U1216, CNRS, Grenoble Institut Neurosciences, 38000 Grenoble, France; 3. Department of Biology, Stanford University, Stanford, CA 94305, USA; 4. Department of Neurology, Beth Israel Deaconess Medical Center, Harvard Medical School, Boston, 02115, MA, USA.; 5. Howard Hughes Medical Institute, Department of Biology, Stanford University, Stanford, CA 94305, USA

**Keywords:** Kinetochore, Dsn1, Ndc80, Mis12, dendritic spine, microtubule, hippocampus, plus-end, cortico-spinal neuron, Drosophila, iNeuron

## Abstract

Kinetochore proteins, long studied for their role in cell division, are also required for the proper postmitotic development of hippocampal and cortical neurons. Proteins of the kinetochore complex were present in axons and dendrites of postmitotic iNeurons where they resided, at least in part, at microtubule plus ends. Conditional deletion of mouse Ndc80 or Dsn1 increased the number of dendritic spines. Loss of any of three kinetochore components (Ndc80, Dsn1, or Mis12) increased microtubule plus-end dynamics. Observations of individual microtubules in C. elegans indicated that Ndc80, the microtubule-binding component of the kinetochore complex, slowed the rate of microtubule growth. The increase in spine number in mammalian neurons correlated with increased microtubule invasion of spines. Both spine number and microtubule invasion phenotypes induced by Ndc80 deletion could be rescued by re-expression of Ndc80, but only if the microtubule-binding region of NDC80 was preserved. We propose that kinetochore proteins act in a complex resembling the mitotic kinetochore in order to stabilize microtubule plus ends and thereby restrain spine invasions and the development of dendritic spines.

## Introduction

The kinetochore protein complex has been studied intensively for its crucial role in cell division. During mitosis, the kinetochore forms a mechanical link between chromosomes and the plus ends of spindle microtubules and thereby mediates chromosome segregation. During this process, intense regulation of the kinetochore helps to guarantee proper chromosome inheritance by the daughter cells (1, 2). Central to the kinetochore are three subcomplexes: the Knl1, Mis12, and Ndc80 complexes, each of which are named after a protein in that subcomplex. Together they are collectively referred to as the KMN complex. Upon completion of mitosis, most kinetochore proteins are degraded and therefore absent from interphase cells (1, 2). Recently however, we and others have found that many kinetochore components are present in invertebrate post-mitotic neurons where they are needed for proper development.

In *C. elegans*, prior work found KMN components to be present in developing sensory neurons. Targeted degradation of these KMN components prevented proper dendrite extension (3). Subsequent work in other *C. elegans* neurons implicated the KMN complex in axon development and fine dendritic branching. Mis-regulation of the actin cytoskeleton was also present in KMN mutant neurons (4, 5).

In *Drosophila*, a mutant screen for abnormal embryonic neuromuscular junctions identified a requirement for mis12 in shaping the synapses. Subsequent tests of mutations and RNAi-mediated knockdown of most of the components of the KMN complex produced the same phenotype – neurite branches that extended too far and failed to form normal synaptic boutons, although vesicles and active zone proteins were still present. Dendrites of embryonic sensory neurons also developed abnormally; instead of avoiding segment boundaries, they overgrew and crossed the boundaries (6). Kinetochore proteins also figure in later stages of development; after the dendrites of a 3^rd^ instar larval sensory neuron were severed, transcripts for these proteins were upregulated and knockdown of those proteins impeded dendrite regrowth (7). This phenotype was accompanied by an increase in microtubule dynamics as monitored by a plus end tracking protein. Moreover, the effect on dendrite regrowth was not only limited to the proteins of the core KMN complex but also occurred upon knockdown of proteins in complexes that regulate the kinetochore: the chromosome passenger and spindle assembly checkpoint complexes. In aggregate, the findings in these invertebrates indicated that the kinetochore is present in the neurites of postmitotic neurons and, because so many components produced similar phenotypes, is probably acting as a complex like the one found on mitotic chromosomes. In addition, the kinetochore proteins present in axons and dendrites likely interact with microtubules – a function potentially akin to their stabilization of spindle microtubules during mitosis (6).

Microtubules are abundant in neurons and provide the structural backbone for maintaining their specialized morphology (8-10). They play crucial roles in neurite growth, neuron migration, maintaining neuron shape, and intracellular trafficking. During development, they are an important source of force to promote axon growth (11). In addition, they influence the development of dendritic spines, the smallest anatomical units where synaptic signals converge and integrate, both biochemically and electrophysiologically (12). Growing evidence indicates that spine density and morphology are highly dynamic, with these structural changes playing a crucial role in synaptic plasticity (13). Although dendritic spines are primarily considered to be actin-based structures, microtubules also figure in spine formation. They transiently invade spines, interact with their actin skeleton, and deliver cargoes to nascent spines (14-18). Because spine number changes with learned experience and changes in spines are associated with intellectual disabilities (19, 20) , the regulation of spine number and plasticity is thought to be key to learning and memory.

In contrast to studies of the kinetochore in invertebrate neurons, much less is known about the function of kinetochore proteins in mammalian neurons. Mis12 protein was detectable, however, in human and rat neurons and knockdown of mis12 in cultured rat hippocampal neurons altered dendritic morphology by increasing the number of filipodia or spine-like protrusions that were present on dendrites (6). To further examine the function of kinetochore proteins in mammalian neurons, we have now turned to mouse genetics and genetic manipulation of human iPSC-derived neurons. We find that kinetochore components are present in cell bodies, axons, and dendrites of mammalian neurons and modulate microtubule dynamics. The absence of kinetochore proteins leads to microtubule mis-regulation, resulting in increased microtubule dynamics. More dendritic spines form both *in vitro* and *in vivo*, which correlates with increased microtubule invasion into the spines. These phenotypes appear to be mediated by the interaction of kinetochore complex, and in particular the microtubule-binding domain of NDC80, with microtubule plus ends.

## Results

### Generation of conditional knockout mice and tagged endogenous proteins in mice and iNeurons

**Figure 1.**
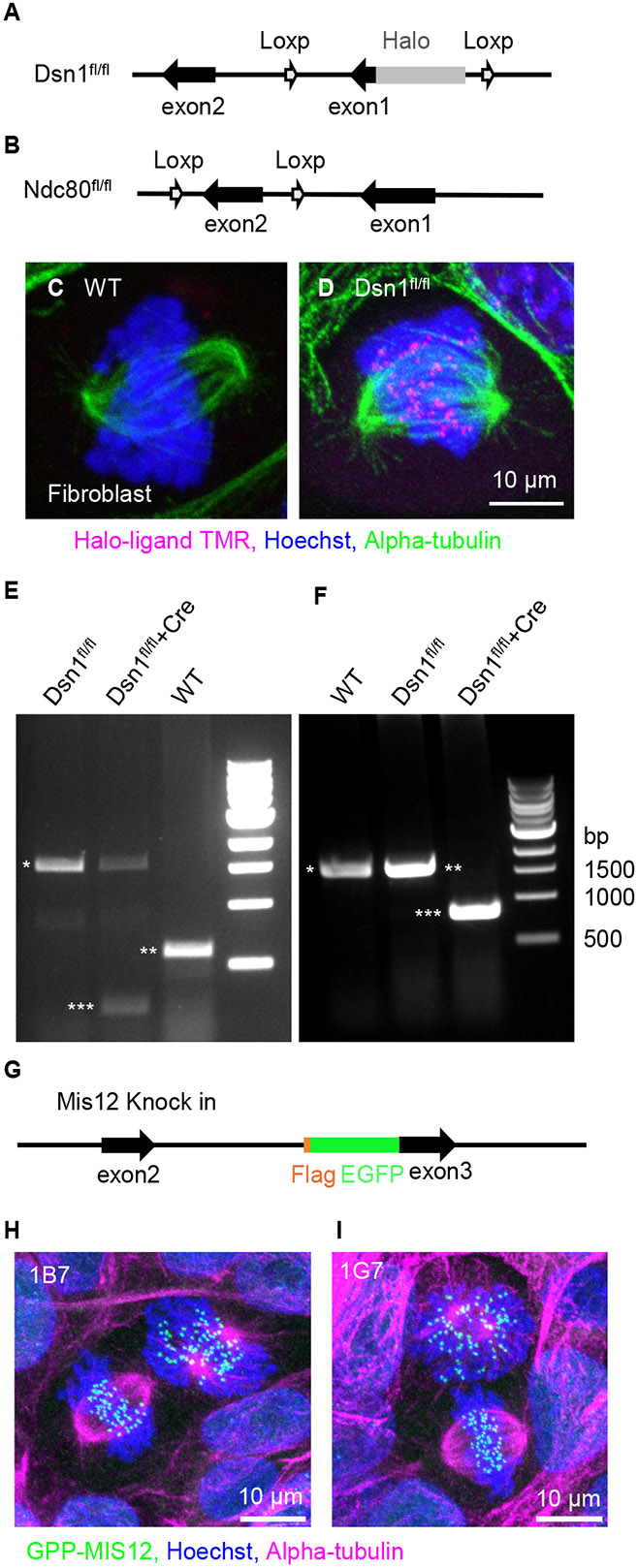
Generation of floxed mice at the Dsn1 and Ndc80 loci, and the EGFP insertion at the MIS12 locus in human iPSC. (A and B) Schematic illustration of the conditional alleles at the Dsn1 (A) and Ndc80 loci (B). For the Dsn1 allele, a Halo tag was also inserted at the Dsn1 N terminus. (C and D) A fibroblast from wildtype (C) and Dsn1 conditional (D) mice stained with the TMR Halo ligand. The magenta puncta indicate Dsn1 properly localized to centromeres of the dividing cell. (E and F) PCR verification of deletion of Dsn1 (E) and Ndc80 (F) exons in cultured neurons after transduction with Synapsin::CRE. The expected bands of PCR products for the indicated genotypes in panel E: Dsn1^fl/fl^ (1593 bp*); Dsn1^fl/fl^ + CRE (379 bp***); wildtype (WT) neurons (610 bp**). The expected bands of PCR products for the indicated genotypes in panel F: wildtype (WT) (1896 bp*); Ndc80^fl/fl^ (1964 bp**); Ndc801^fl/fl^ + Cre (848 bp***). The incomplete removal of the Dsn1 exon in E lane Dsn1^fl/fl^ + CRE may be due, in part, to the presence of non-neuronal cells not expressing Cre. (G) Schematic illustration of the insertion of a EGFP and Flag tag into the Mis12 locus in iPSC lines. (H and I) Examples of individual clones (1B7 and 1G7) carrying the EGFP insertion at the Mis12 locus, with the expected staining of mitotic chromosomes.

To explore kinetochore function in post-mitotic neurons, while circumventing the requirement of the kinetochore proteins for cell division during development, we generated mice carrying floxed alleles of Dsn1 or Ndc80 (Figure 1A, 1B and Supplemental Figure 1). At the Dsn1 locus, in addition to inserting the loxP sequences, we also inserted a Halo tag at the N-terminal of the protein to aid in its localization (Figure 1 A). The function of the tagged DSN1 protein was confirmed by the viability of the homozygous mouse, the ability of cultured fibroblasts to divide correctly, and the expected localization of the tagged protein to centromeres of dividing fibroblasts (Figure 1 C and 1 D). We also verified the efficacy of the deletion of Dsn1 and Ndc80 by Cre recombinase in cultured hippocampal neurons by expressing Cre or inactive Cre (ΔCre) under control of a synapsin promoter and PCR amplification of the genomic region 5 days later. (Figure 1E and 1F). As a further aid in the localization of kinetochore mutations, we also inserted an EGFP tag at the N-terminus of MIS12 in a human iPSC line and obtained one line with a heterozygous insertion of the tag (1D7) and two with homozygous insertions (1B7, 1G7) (Figure 1G and Supplemental Figure 1C and 1D). The correct insertions were verified by PCR amplification and sequencing (Supplemental Figure 1D). Proper function of the EGFP-MIS12 protein was confirmed by the viability of the iPSC line and the expected localization of EGFP-MIS12 at the centromeres of dividing iPSCs (Figure 1H and 1I). Probing a western blot of lysates of the iPSC lines with an antibody to human MIS12 showed that EGFP-MIS12 protein expresses at comparable levels to endogenous MIS12 in both iPSCs and Ngn2-differentiated iNeurons (Supplemental Figure 1E). The inserted tags on DSN1 and MIS12 proved particularly useful for immunolocalizations because few of the commercial antibodies against kinetochore proteins were effective on postmitotic mouse neurons or human iNeurons.

### Loss of Dsn1 or the microtubule-binding region of NDC80 increases dendritic spines.

**Figure 2.**
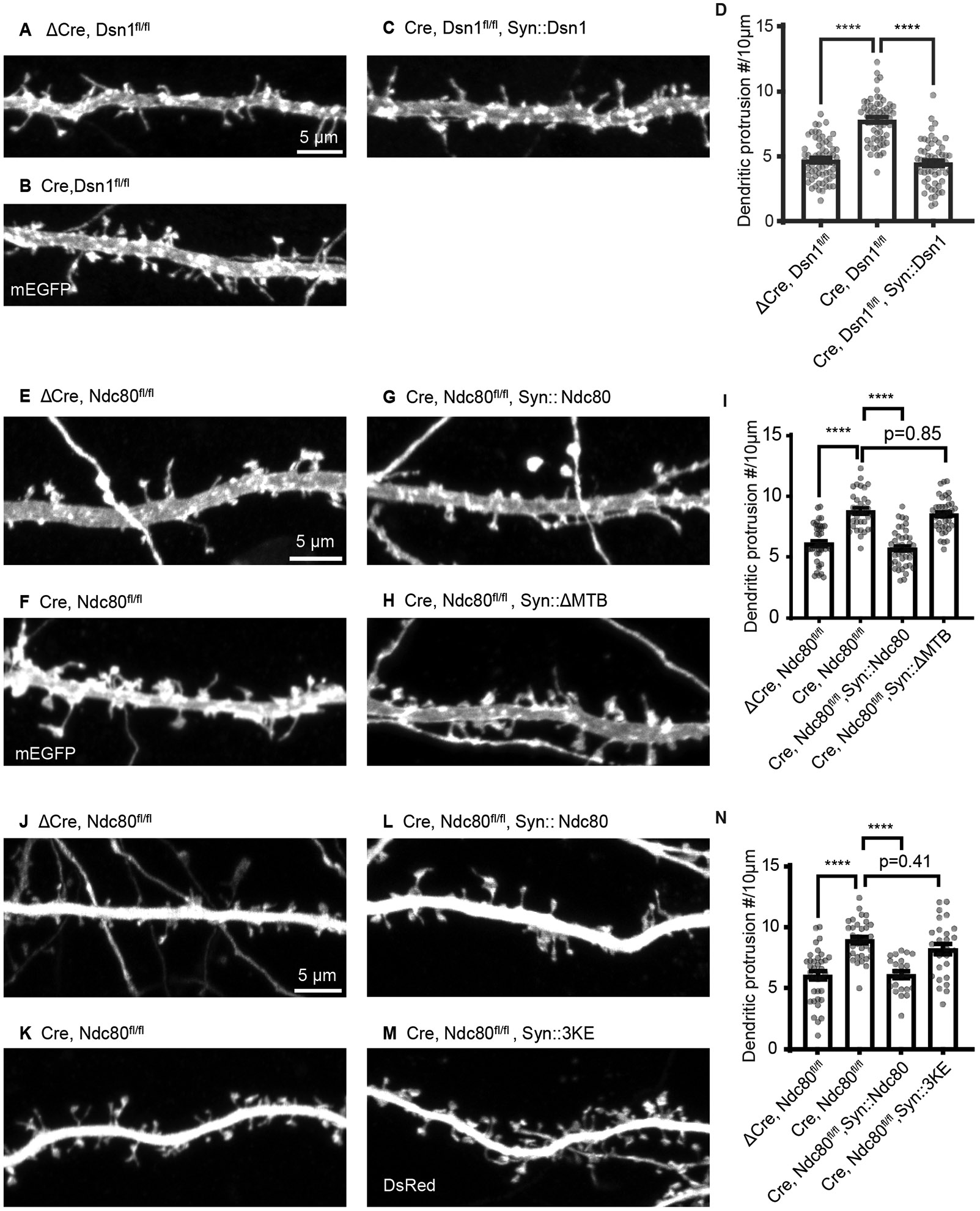
Knockout of kinetochore components increased dendritic spines in cultured hippocampal neurons. (A-C) Hippocampal neurons from Dsn1^fl/fl^ mice were infected on DIV11 with lentivirus expressing inactive ΔCre (A), Cre (B) or Cre with a rescue virus expressing DSN1(C). The neurons were transfected with plasmid expressing membrane-bound GFP on DIV13, fixed on DIV17, and dendritic spines were quantified according to the GFP signal (D). (E-H) Hippocampal neurons from Ndc80^fl/fl^ mice were infected on DIV8 with lentivirus expressing inactive ΔCre (E), Cre (F), Cre with rescue virus expressing wildtype NDC80 (G) or Cre with a rescue virus expressing microtubule-binding deficit NDC80 (ΔMTB-NDC80) (H) Neurons were transfected with plasmid expressing membrane bound GFP on DIV10, fixed on DIV14, and dendritic spines were quantified according to the GFP signal (I). (J-M) Hippocampal neurons from Ndc80^fl/fl^ mice were transfected on DIV10 with plasmids expressing inactive ΔCre (J), Cre (K), Cre plus an NDC80 rescue construct (L) or Cre plus NDC80 with mutated microtubule binding sites (M), and membrane bind GFP. (N) Quantification of dendritic spines. In (D, I, N), graphs indicate mean ± SEM and each dot represents one neuron. One-way ANOVA with Tukey's multiple comparisons test was performed and adjusted p value are shown. **** p < 0.0001.

We cultured hippocampal neurons from E18 embryos carrying the floxed alleles and infected them with lentivirus expressing either Cre or the inactive ΔCre under control of a synapsin promotor 9 days after plating (DIV9). The neurons were transfected two days later with a plasmid expressing a membrane-localized GFP and neuronal morphology was visualized on DIV14. As we had previously observed with shRNA knockdown of Mis12 in hippocampal neurons (6), the Cre-expressing Dsn1^fl/fl^ neurons had more spine-like or filipodia-like protrusions per 10μm than the ΔCre controls (Figure 2 A, B and D). We quantified these protrusions by selecting a length of dendrite at a fixed distance from the soma (see Methods). The specificity of the phenotype was confirmed by restoring normal spine counts with rescue constructs that expressed DSN1 under control of a synapsin promotor (Figure 2 C and D). The same CRE-dependent phenotype occurred in cultures of Ndc80^fl/fl^ neurons (Figure 2 E-G and I). Dendritic spines are actin-rich structures that, when mature, contain the postsynaptic density marker PSD95 and are adjacent to the presynaptic marker Synapsin (21, 22). Staining of the Ndc80 deleted neurons indicated that the dendritic protrusions shared these features of dendritic spines (Figure S2). The phenotype was not restricted to hippocampal neurons; Cre-mediated deletion of Ndc80 or Dsn1 in cultured cortical neurons yielded a similar increase in dendritic spines (Figure S3).

NDC80 binds directly to microtubules during cell division and is the component principally responsible for attaching the kinetochore complex to the mitotic spindle. Previous studies have mapped the regions of NDC80 that interact with microtubules and residues within those regions that are essential for microtubule binding (23, 24). Based on this information, we made two microtubule-binding deficient forms of NDC80 to test the significance of the microtubule-binding function of NDC80 for the spine-formation phenotype by determining if they could restore proper spine counts after Ndc80 deletion. The first mutant NDC80-3KE replaced with glutamates three critical lysines in the microtubule-binding site (K89, K115, and K166). The second construct, in addition to the three amino acid changes, also lacked residues 1-79 (ΔMTB-NDC80). Unlike intact NDC80, neither of the mutated constructs could rescue the effect of Ndc80 deletion (Figure 2F, 2H 2I and 2J-2N). Thus, although dendritic spines are fundamentally actin-rich structures, the microtubule-binding property of NDC80 is necessary to regulate the number formed. That Ndc80 and Dsn1 deletion yielded the same phenotype as we had observed upon knockdown of Mis12 (6) indicates that these proteins likely act together as parts of a single kinetochore complex, much as they do during mitosis.

### Loss of kinetochore proteins increases dendritic spines in vivo.

**Figure 3.**
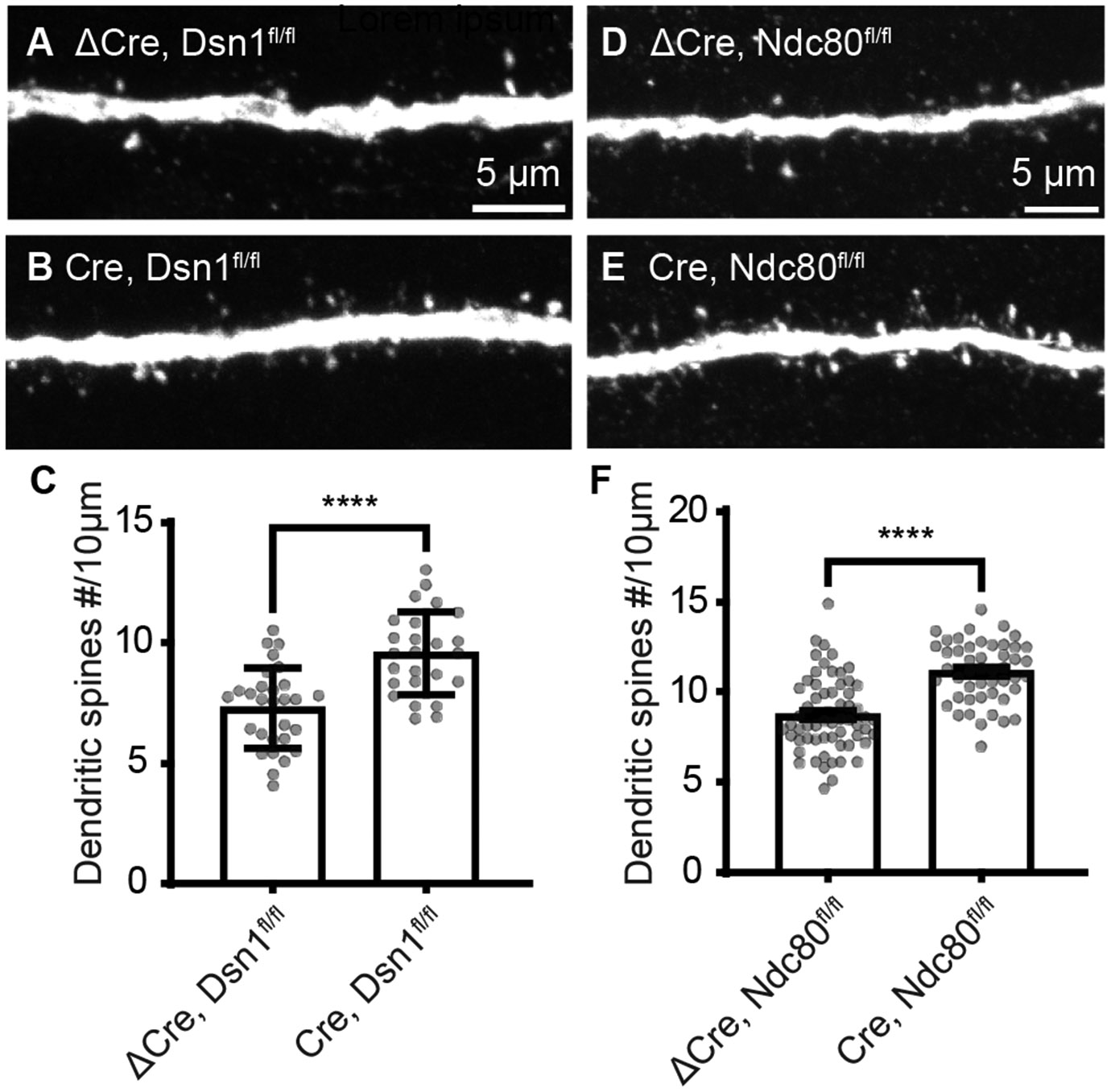
Knockout of kinetochore components increased dendritic spines in mouse cortical neurons. (A and B) Representative images of dendrites of P45 cortical neurons from Dsn1^fl/fl^ mice that were infected by inactive ΔCre (A) or Cre (B) expressing virus on P7. (C) quantification of dendritic spine density on these neurons. (D and E) Representative images of dendrites of P45 cortical neurons from Ndc80^fl/fl^ mice that were infected by inactive ΔCre (D) or Cre (E) expressing virus on P7. (F) quantification of dendritic spine density on these neurons. Graphs indicate mean ± SEM; each dot represents one neuron. Welch’s t test was performed. **** p < 0.0001.

To investigate *in vivo* the role of kinetochore components in postnatal brain development, we injected retrovirus expressing Cre or ΔCre into P7 mouse spinal cords to delete Ndc80 or Dsn1 in corticospinal neurons of the floxed alleles. The Cre and ΔCre constructs also had a T2A sequence followed by GFP so that GFP expression would mark infected neurons and enable their tracing. 38 days after the injection, we cut sections of whole brains, stained slices with anti-GFP and quantified dendritic spines at a fixed distance from the soma of the labelled neurons. Loss of Ndc80 or Dsn1 increased the number of dendritic spines relative to control-injected mice (Figure 3A-3F), consistent with what had occurred in cultured neurons. Collectively, these findings demonstrate that deletion of Ndc80 or Dsn1 alters dendritic spine density both *in vitro* and *in vivo*, supporting a novel role for kinetochore components in shaping the architecture of the synaptic compartment.

### Kinetochore protein localization in postmitotic neurons

**Figure 4.**
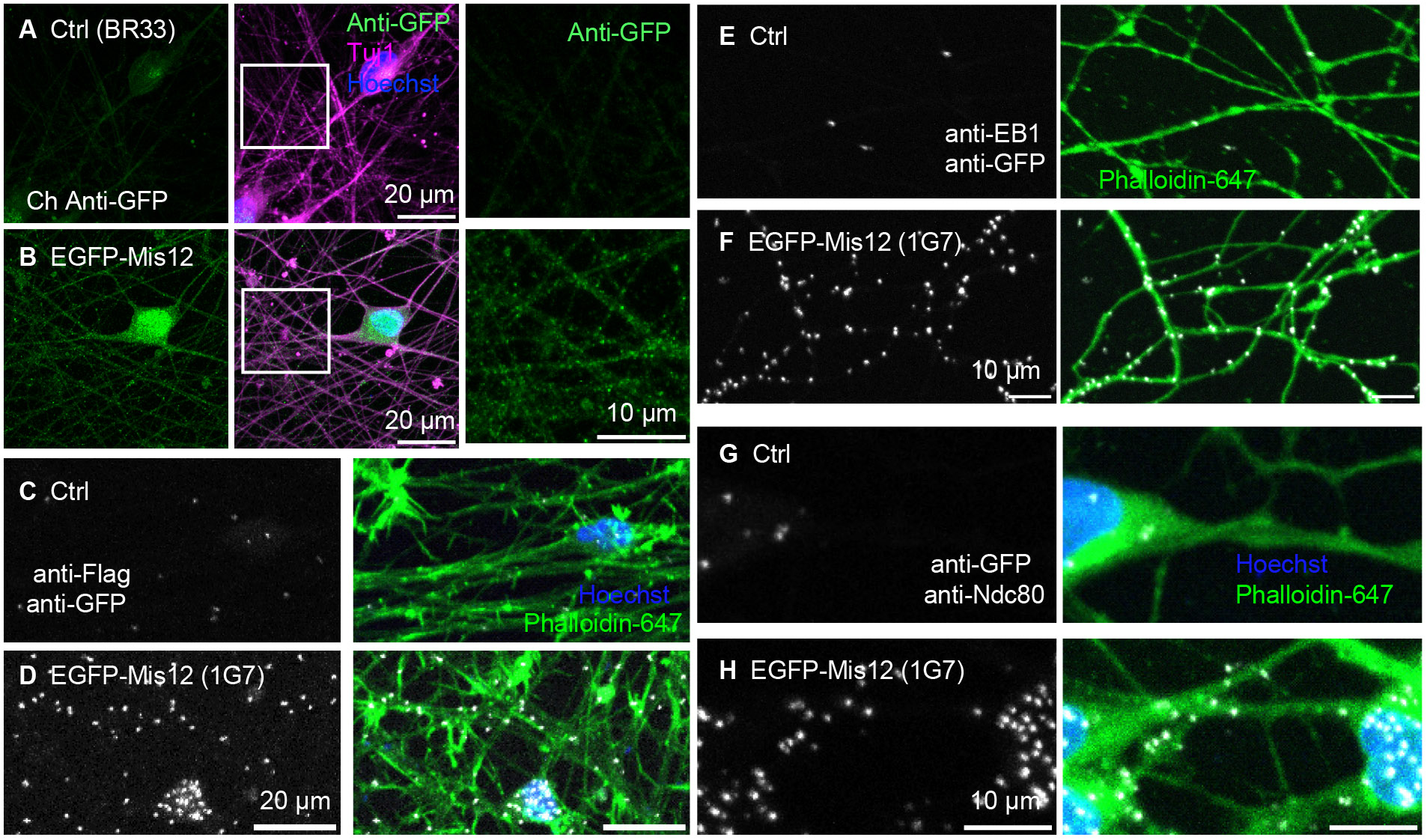
Localization of the kinetochore protein MIS12 in iPSC-derived neurons. (A and B) Localization of FLAG-GFP-Mis12 in iNeurons. Wildtype (A) and FLAG-EGFP-knock in (B) iNeurons were labeled with anti-GFP. Left and middle panels of A and B show anti-GFP signal (green) and anti-GFP overlayed with anti-Tuj1 (magenta) and Hoechst dye channels (blue). The boxed areas are shown at higher magnitude in the righthand panels; (C-H) The proximity ligation assay (PLA) was used for localization of MIS12 in iNeurons. (C and D) FLAG-EGFP-MIS12 localization by PLA reaction between anti-Flag and anti-GFP in D14 (14 days after differentiation) iNeurons from wildtype (C) and FLAG-EGFP-knock in (D) lines. (E and F) Colocalization of FLAG-EGFP-MIS12 with EB1 by PLA reaction between anti-GFP and anti-EB1 in wildtype (E) and FLAG-EGFP-knock in (F) D11 iNeurons. (G and H) Colocalization of FLAG-EGFP-MIS12 and NDC80 by PLA reaction with anti-NDC80 and anti-GFP in wildtype (G) and FLAG-EGFP-knock in (H) D6 iNeurons. PLA reactions appear as white puncta; neurites were visualized with phalloidin (green) to label F-actin and nuclei are stained with Hoechst dye (blue).

Kinetochore proteins localize to chromosomes during cell division, but their localization in postmitotic neurons is unclear. We attempted without success to localize several components of the kinetochore in cultured mouse hippocampal neurons using commercial antibodies that are commonly used to study mitotic cells (mouse anti-HEC1, clone 9G3; rabbit anti-KNL1, ab70537; rabbit anti- CASC5, A13108). However, whereas kinetochores are densely concentrated at the centromeres of metaphase chromosomes, which facilitates their visualization, they were not persuasively detectable over background in postmitotic neurons with these same antibodies, presumably due to sparser distribution. We therefore differentiated EGFP-MIS12 knock-in iPSCs into cortical-like neurons (iNeurons). The GFP-fluorescence was not detectable over background, but immunostaining with anti-GFP detected EGFP-MIS12 in both cell bodies and neurites (Figure 4A, 4B). At high magnification, individual puncta in the neurites were easily discerned from background and were not present in iNeurons differentiated from the parent BR33 line. Because the MIS12 knock-in had both EGFP and FLAG tags, we could enhance the signal to noise ratio by using antibodies to both tags in a proximity ligation assay where only the close juxtaposition (less than 40 nm) of both tags could yield the reaction product (Figure 4C and 4D). The proximity ligation reaction produced puncta in the EGFP-MIS12 iNeurons with almost none in the BR33-derived iNeurons and confirmed the presence of the protein in somas and neurites.

In mitosis, the kinetochore associates with the plus ends of microtubules. We therefore used the proximity ligation assay to look for the association of EGFP-MIS12 (with anti-GFP) and a plus-end binding protein (with anti-EB1). A signal was detected in neurites and cell bodies of the EGFP-MIS12 iNeurons that was not present in the parent BR33 line (Figure 4E and 4F) and colocalized with tyrosinated tubulin (Figure S4C and D). Thus at least some of the MIS12 colocalized with microtubule plus ends. To examine the possible relationship of NDC80 with EB1-containing plus ends, we expressed by Lentivirus a GFP-tagged version of either wild-type NDC80 or NDC80 that lacked the microtubule-binding region (ΔMTB-NDC80) in ndc80^fl/fl^ neurons and performed the proximity ligation assay between the GFP tag and EB1 (Figure S5). The assays were performed in Ndc80^fl/fl^ neurons co-infected with Cre-mCherry virus to eliminate endogenous NDC80. Neurons expressing wildtype NDC80 had more PLA signal than those with ΔMTB-NDC80 or uninfected neurons. Thus, NDC80 also can localize to microtubule plus ends and in a manner dependent on its microtubule-binding domain.

The similarity of the phenotypes obtained by knock-out or knockdown of Ndc80, Dsn1, and Mis12, and the presence of both NDC80 and MIS12 at plus ends, suggested that the kinetochore proteins function together as a complex in neurons, as they do in dividing cells. Therefore, to ask whether MIS12 colocalizes with other kinetochore components, we used anti-GFP and anti-human NDC80 antibody in a proximity ligation assay. The presence of puncta of reaction product in EGFP-MIS12 iNeurons but not in BR33 iNeurons indicated that MIS12 and the NDC80 can co-localize with each other in neuronal cell bodies and neurites (Figure 4G and 4H). The assay reaction product was present in iNeurons 6 day and 20 days after differentiation, indicating that the complexes were unlikely to be residue from mitotic events prior to differentiation (Figure S4A and S4B). Together, these results reveal the persistence of kinetochore proteins beyond the window of mitotic activity, underscoring their relevance to postmitotic neurons and a likely role at microtubule plus ends.

### Loss of kinetochore proteins increases the dynamics of neuronal microtubules

**Figure 5.**
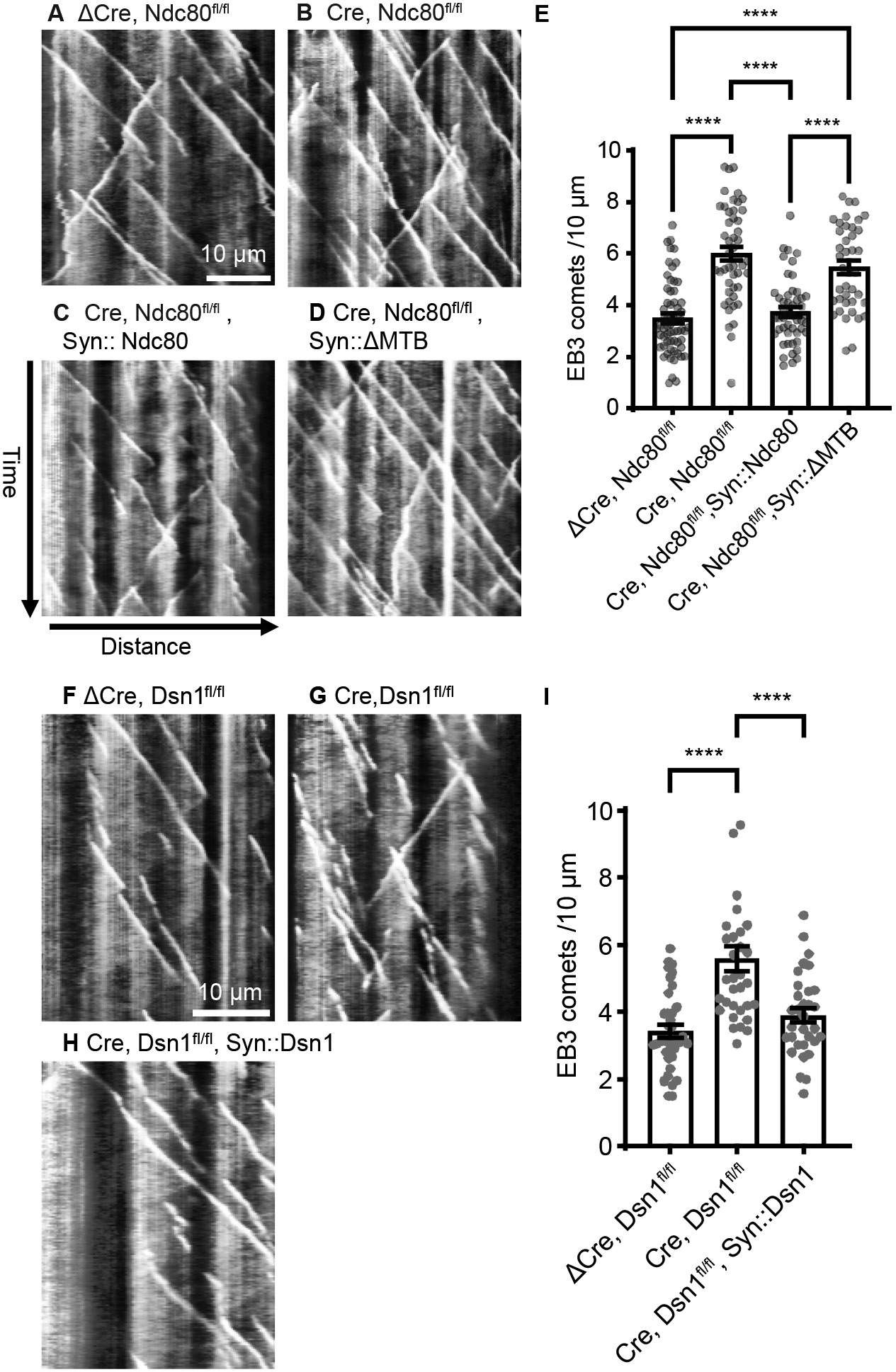
Increased numbers of growing microtubule plus ends upon loss of Ndc80 or Dsn1. (A-E) Ndc80^fl/fl^ neurons were infected with inactive ΔCRE (A), CRE (B), CRE and a virus expressing Ndc80 (C) or CRE and a virus expressing ΔMTB-NDC80(D) and also with EB3-tdTomato. Representative kymographs of EB3 signals are shown with comets representing growing microtubule plus ends and anterograde movement is towards the right. Each kymograph represents 3 minutes of recording from a length of dendrite and the frequency of those comets per length of neurite is quantified in (E). (F-I) Dsn1^fl/fl^ neurons were infected with inactive CRE (F), CRE (G), or CRE and a virus expressing Dsn1 (H) and EB3-tdTomato comets quantified from the resulting kymographs (I). Graphs indicate mean ± SEM; each dot represents one neuron. One-way ANOVA with Tukey's multiple comparisons test was performed and adjusted p value are given in the figure. **** p < 0.0001, *** p < 0.001.

Microtubules are enriched in neurons and highly dynamic, especially during development. The percentage of microtubules that are stable increases in mature axons after they have reached their targets (25). At their plus ends, microtubules can grow by the addition of tubulin dimers or shrink by sudden depolymerization (26), and the position of growing plus ends can be monitored by live-imaging of plus-end tracking proteins such as EB1-GFP or EB3-GFP. Each actively extending microtubule thus gives rise to a “comet” that reflects the advancing plus end. To investigate how kinetochore knockout affects neuronal microtubules, we expressed EB3-tdTomato in hippocampal neurons that were isolated from floxed Ndc80 mouse embryos. The movements of EB3-tdTomato puncta were recorded in dendrites for 3 minutes and the movies thus generated were subsequently analyzed by kymography. We found that the number of comets significantly increased in Ndc80 knockout neurons compared to control neurons (Figure 5A, B, E). This increase in EB3-comets was rescued by viral expression of wildtype Ndc80, but not by the microtubule-binding deficient form of Ndc80 (ΔMTB-Ndc80) (Figure 5C, D, E). Thus, the microtubule-binding property is crucial for the regulation of microtubule dynamics by Ndc80. A similar increase in growing plus ends was found in Dsn1 knockout neurons expressing EB3-tdTomato (Figure 5F-I). Furthermore, we checked how Mis12 knockdown affects microtubule dynamic by transfecting rat hippocampal neurons with EB3-EGFP and shRNAs targeting Mis12. Expression of shRNAs targeting two different regions of Mis12 gave similar results (Figure S6A-C). Thus, multiple components of the kinetochore, including the microtubule-binding regions of Ndc80 are involved in limiting the growth of microtubule plus ends. An increase in microtubule plus ends can result either from an overall increase in microtubule number or from enhanced dynamics within a constant microtubule population. To distinguish between these possibilities, we assessed total microtubule content by immunostaining ΔCre and Cre-expressing Ndc80^fl/dl^ hippocampal neurons with anti-βIII-tubulin. Microtubule abundance appeared unchanged as judged by immunostaining for tubulin (Figure S7). Thus, the observed increase in plus end comets is more likely due to altered microtubule dynamics than to an increase in microtubule number.

**Figure 6.**
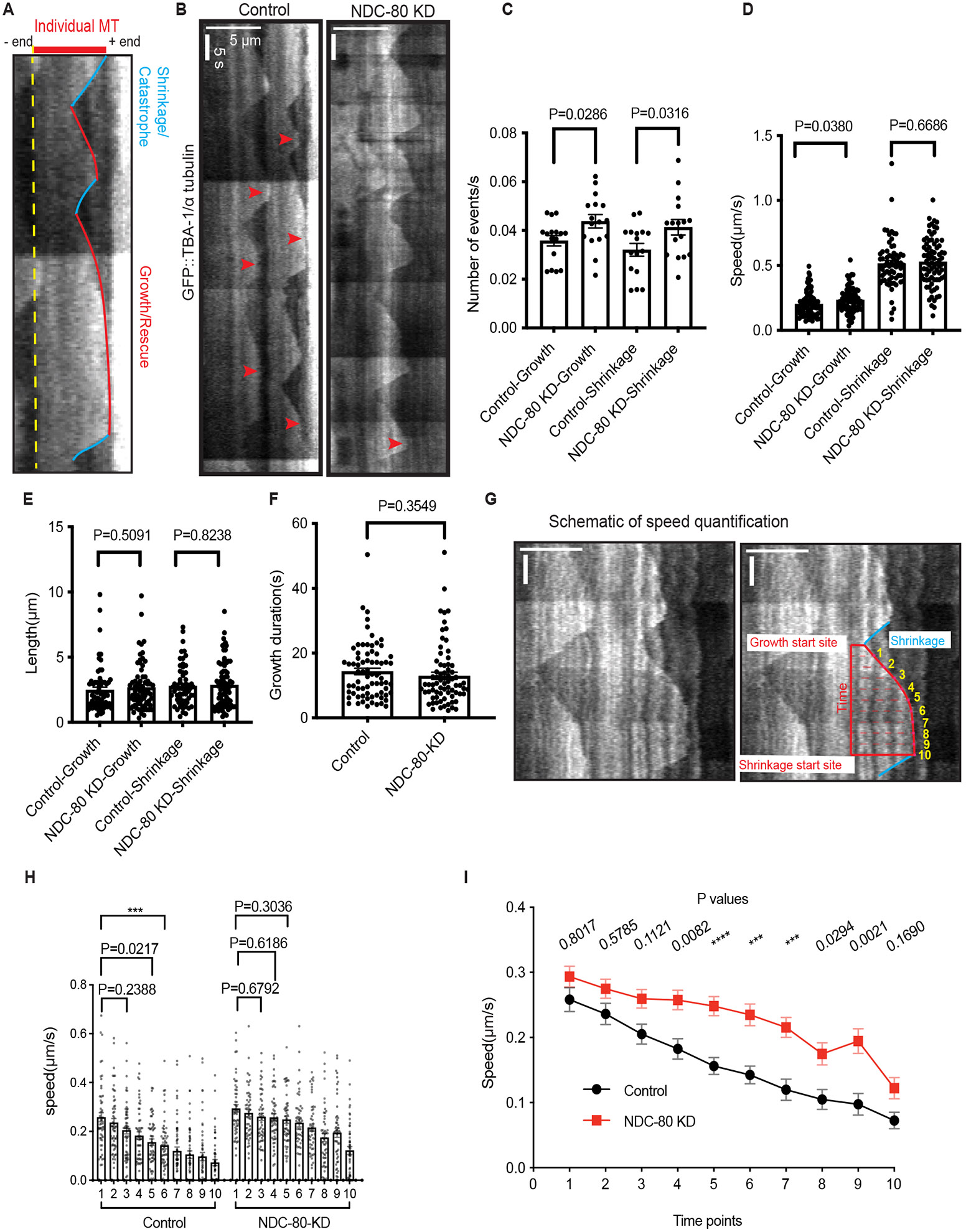
Ndc80 modulates microtubule dynamics in *C. elegans.* (A) Representative kymograph to illustrate the measurement of MT dynamics in PVD neurons. Blue lines: MT shrinkage/catastrophe events; red lines: MT growth/rescue events; yellow dashed line, MT minus end. The kymograph shows a zoomed-in region from panel B. (B) Representative kymographs of MT dynamics in control and NDC-80 knock-down animals. The red arrowheads mark the slowing down or pausing events before the starting of catastrophe. (C) Quantification of MT growth or shrinkage frequency. Control: n=16 MTs from 8 animals; NDC-80 KD: n=16 MTs from 12 animals. (D) Quantification of MT growth or shrinkage speed. Control: n=74 (growth), n=65 (shrinkage); NDC-80 KD: n=85 (growth), n=77 (shrinkage). (E) Quantification of MT dynamic length. Control: n=66 (growth), n=61 (shrinkage); NDC-80 KD: n=73 (growth), n=77 (shrinkage). (F) Quantification of MT growth duration. Control: n=72; NDC-80 KD: n=73. (G) Schematic of MT growth speed quantification across 10 equal segments. (H) Quantification of MT growth speed at each segment using the approach indicated in G. The statistical comparisons within a single genotype demonstrate the change in speed as the growth event progresses(I) Quantification of MT growth speed changes from the beginning to end of growth for a comparison of MT growth properties between control and NDC-80 KD neurons. Control: n=55; NDC-80 KD: n=51. For C-F), P values were calculated by Welch’s t test; for H-I), P values were calculated by Brown-Forsythe and Welch ANOVA test. Each dot in graphs represents an individual microtubule (C, D) or growth or shrinkage event (E, F, and H).

To examine a wider range of kinetochore components, we returned to *Drosophila* (6) and used the sensory neuron-specific ppk-Gal4 driver to express EB1-GFP together with RNAi against mis12, ndc80 or spc105R. The number of EB1-GFP comets per micron of dendrite was increased in each case compared to the control (Figure S6D and E). To look closely at the dynamics of microtubule plus ends, we turned to the primary dendrites of PVD neurons in *C. elegans*. In the posterior regions of these dendrites, the microtubules are uniformly plus-end out and the number of microtubules is so low that individual microtubules can be visualized when one of the main alpha-tubulins is GFP-labeled (GFP::TBA-1) (27). Consequently, in time-lapse imaging, both the growth phase and shrinkage of individual microtubules can be characterized without recourse to a plus-end binding protein. Each dynamic microtubule presents a saw-toothed profile in which the dynamic region repeatedly grows and shrinks (Figure 6A). In NDC-80 knock down animals, both rescue and catastrophe frequency were increased (Figure 6B and C), Moreover, while there was an increase in the speed of growth for the individual microtubules in the NDC-80 knock down animals, the speed of their shrinkage was unaffected (Figure 6D). The individual microtubule growth length and growth duration were not changed (Figure 6E and F). To examine more closely the growth dynamics, the growing phase of each microtubule was subdivided into 10 equal time segments and the growth speed calculated for each segment. In control dendrites, the rate of growth steadily declined as the plus end advanced. In the NDC-80 knock down mutants, however, the rate of growth was more consistently rapid until the final time segments (Figure 6G-I). The difference in growth rates in NDC-80 knock down mutants suggests that NDC-80 might function at the growing plus ends to restrain the rate of growth. That microtubule dynamics are increased by kinetochore mutations across mouse, rat, *Drosophila*, and *C. elegans* neurons suggest a conserved role for kinetochore components in limiting microtubule dynamics and stabilizing plus ends in postmitotic neurons.

### Ndc80 knockout Increases microtubule invasion of dendritic spines.

Kinetochore components might limit the growth of dendritic spines by regulating the entry of dynamic microtubules into the spines. We therefore tested the effect of Ndc80 deletion on the rate of microtubule invasions of spines. Cultured hippocampal neurons from Ndc80^fl/fl^ mice were infected at DIV7 with a bicistronic lentiviral vector encoding both the actin marker LifeAct-RFP, which strongly highlights spines, and the plus-end tracker EB3-YFP. Cells were subsequently infected with Cre- or ΔCre-encoding virus, and with or without rescue constructs, and left for 6 days to allow for full depletion of endogenous Ndc80. At DIV18, high-resolution videos of EB3-YFP and LifeAct-RFP were simultaneously acquired, revealing both the comet-like motion of EB3 puncta and the changing shape of spines (Videos S1-S4). Spine-invading microtubules were visible as thin EB3 comets transiently exiting the dendritic shaft and entering the actin-rich spines.

**Figure 7:**
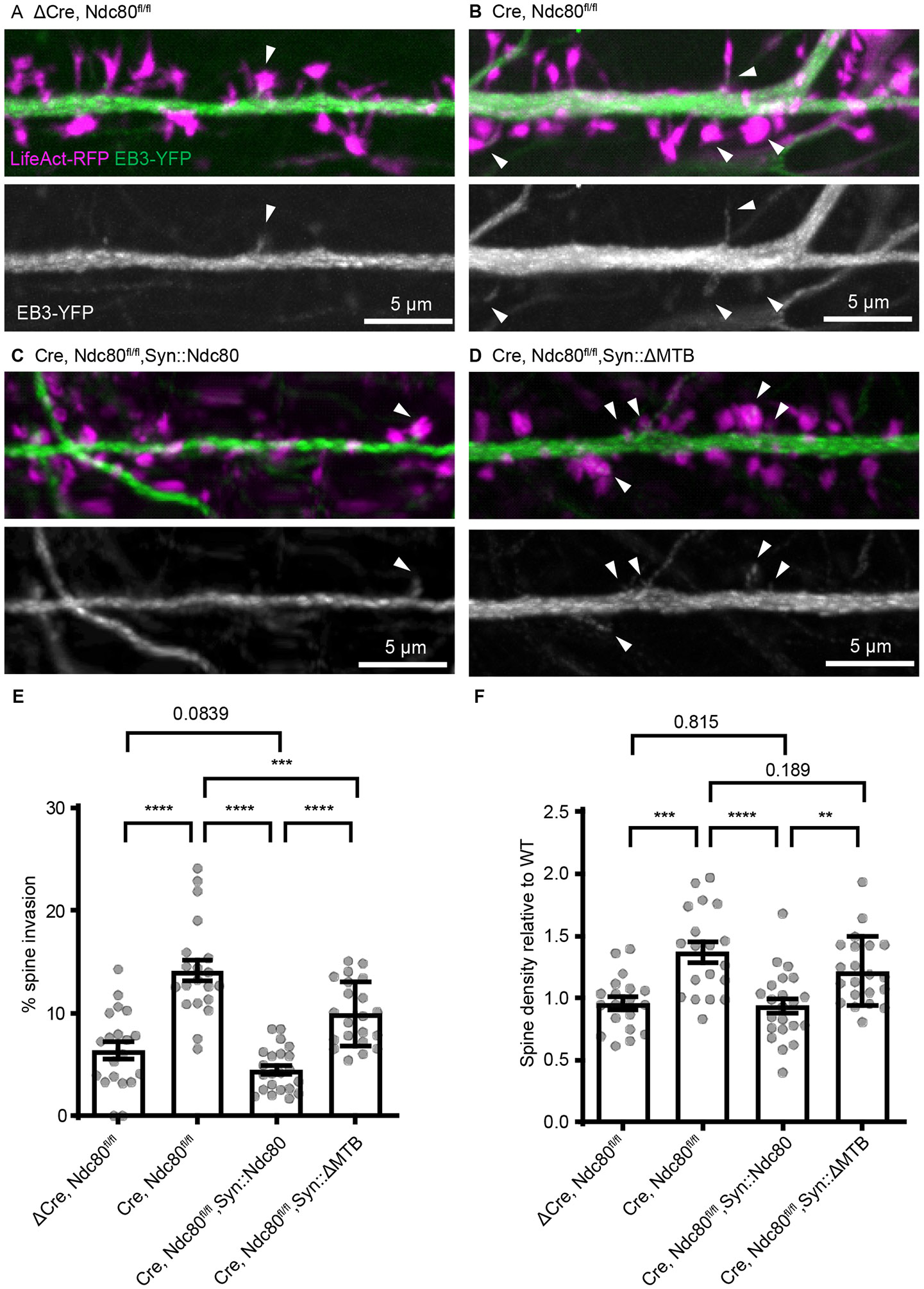
Ndc80 interaction with microtubules modulates their invasion of dendritic spines and regulates spine density. (A-D) Representative maximal intensity projections of time-lapse confocal images showing dendritic segments of 18 DIV Ndc80^flox/flox^ hippocampal neurons expressing both LifeAct-RFP (magenta) and EB3-YFP (green upper panels; gray lower panels). Ndc80^flox/flox^ neurons were infected with lentivirus encoding defective ΔCre (A), Cre (B), Cre and wild-type Ndc80 (C) or CRE and ΔMTB-NDC80 (D). White arrows indicate microtubules invading dendritic spines. Scale bar: 5 μm. (E) Percentage of spines invaded by microtubules in Ndc80^flox/flox^ neurons from 5 minutes of time-lapse images as in (A-D). Data represents SEM; n = 20, 20, 22 and 21 for neurons expressing ΔCre, Cre, Cre + Ndc80 and Cre + Ndc80 DMTB respectively, from at least three different neuronal cultures. One-way ANOVA with Holm-Sidak's multiple comparisons test. **** p < 0.0001, *** p < 0.001 and adjusted p value is represented in the figure. (F) Spine density of Ndc80^flox/flox^ neurons of each condition. Values were normalized to the mean of the control cells. Data represent mean ± SEM; n = 19, 19, 22 and 21 neurons expressing ΔCre, Cre, Cre + Ndc80 and Cre + Ndc80 DMTB respectively, from at least three different neuronal cultures. One-way ANOVA with Holm-Sidak's multiple comparisons test. **** p < 0.0001, *** p < 0.001, ** p < 0.01 and adjusted p value are represented in the figure.

The number of spine invasions observed scaled linearly with the duration of the observation period, We therefore compared across conditions the fraction of spines that were invaded during 5 minutes. The fraction of invaded spines was more than two-fold higher in neurons transduced with active Cre than in controls with ΔCre (Figure 7 A, B, E). Rescue by exogenous wild-type NDC80 brought this invasion rate back to control level, confirming the specificity of the Cre-mediated deletion phenotype (Figure 7 C, E). In contrast, expression of the NDC80 ΔMTB mutant was significantly less effective than wildytpe NDC80 in restoring the normal incidence of spine invasions (Figure 7 D, E). Taken together, these results indicate that, in mature neurons, NDC80 restrains the frequency of spine entries by microtubules in a process that involves Ndc80 binding to microtubules.

Counting the LifeAct-labelled spines in the same neurons revealed a 38% increase in spine density in Ndc80 deleted neurons compared to controls, as in Figure 2, and this increase was reversed by expression of wildype but not ΔMTB Ndc80 (Figure 7 A-D, F). Thus, the inhibitory effect of NDC80 on spine counts parallels that on microtubule invasions of spines. The rate of microtubule invasions may therefore be a limiting factor for the growth or maintenance of spines and under control of a direct interaction between NDC80 and microtubules.

From the same neurons, parameters of microtubule dynamics were measured (Figure S8). In agreement with Figure 5, the density of EB3 comets was about two-fold higher in neurons transduced with Cre than in controls with ΔCre (Figure S8A, B), confirming that dynamic microtubules become more abundant in the absence of NDC80. Additionally, and consistent with augmented intrinsic dynamics, the catastrophe frequency, the microtubule nucleation or rescue rate and microtubule growth rate (Figure S8A, C, D, E) were significantly increased in neurons with Ndc80 deletion. On the other hand, Ndc80 deletion did not detectably affect either the comet growth length or the proportion of anterograde vs. retrograde comets (Figure S8F, G.) Unexpectedly, parameters of microtubule dynamics in the dendritic shaft did not appear to be effectively restored by transduction with the wild-type NDC80 construct (Figure S8B-E). The discrepancy between rescue of spine invasions and microtubule dynamics in the dendritic shaft remains unresolved. Potentially, because different microtubule populations exist in dendrites (28), local regulation of a specific subpopulation that participates in spine invasion may underlie how Ndc80 limits spine growth.

## Discussion

We have studied the function of kinetochore proteins in post-mitotic mammalian neurons and observed that the deletion of two kinetochore components (Dsn1and Ndc80) increased the formation of dendritic spines (Figure 2). This phenotype was apparent in both hippocampal and cortical neurons in culture (Figure 2 and Figure S3) and also in cortico-spinal neurons *in vivo* (Figure 3). These phenotypes appear to arise from a change in microtubule dynamics as they were accompanied by an increase in growing plus ends (Figure 5) and an increase in plus-end invasions of dendritic spines (Figure 7). Moreover, proper regulation of plus-end spine invasions and of spine number required the microtubule-binding domain of NDC80 (Figures 2 and 7). Consistent with these observations, kinetochore proteins were present in close proximity to the plus-end associated protein EB1 (Figure 4) and the rate of plus-end growth was increased in both hippocampal and *C. elegans* dendrites when Ndc80 was absent. We propose, as the most parsimonious account of these results, that Ndc80 and the kinetochore proteins exert inhibitory tone on dendritic microtubule dynamics, limiting plus-end growth and entries into existing spines, and thereby regulating the maintenance or maturation of spines.

During mitosis, kinetochore components function together as a complex and loss of an individual component suffices to disrupt cell division (1). Although in mammalian neurons we have only disrupted Dsn1, Ndc80 and Mis12, it is likely that many and perhaps all the KMN kinetochore components are functioning together as well. The proximity ligation assay determined MIS12 and NDC80 to be in close proximity (<40nm) in cell bodies and neurites (Figure 4 and S4), suggesting that at least a portion of these proteins were co-assembled into a complex. These observations are consistent with those we and others have made in *Drosophila* and *C. elegans* (3, 6, 7). In *Drosophila*, components in each of the major kinetochore subcomplexes are needed for proper formation of neuromuscular junctions and sensory dendrites (6) and sensory dendrite regeneration (7). In *C. elegans,* degradation of multiple kinetochore proteins disrupted dendrite extension, dendrite branching, and sensory neuron patterning (3-5). Moreover, the normal localization of Ndc80 and Spc25 in *Drosophila* neuropil was disrupted in *mis12* mutants (6). The presence of kinetochore complexes in mammalian cell bodies and neurites is unlikely to arise from perdurance of mitotic KMN complexes. MIS12-NDC80 associations were present 20 days after iNeuron differentiation (Figure S4). Most persuasively, a phenotype was evoked by *in vivo* injection of Cre-expressing virus to delete either Ndc80 or Dsn1 from postnatal corticospinal neurons whose axons had already reached their targets (Figure 3). In aggregate, we conclude that a complex of kinetochore proteins is present in and necessary for postmitotic neuronal development.

One clear distinction between kinetochore complexes in mitosis and in neurons is their localization. In dividing cells, the endogenous proteins are intensely concentrated and easy to observe at centromeres (as in Figs 1D, 1H and 1I), whereas they are extremely difficult to localize with the same reagents in neuronal cultures. Nevertheless, having tagged the endogenous MIS12 protein with EGFP and FLAG, we could find immunoreactive puncta in neurites and cell bodies (Figure 4A and B) and the signal to noise ratio could be improved by proximity ligation assay (Figure 4C-H). These signals may arise from just one or a few copies of each of the assayed proteins at a given plus-end and their sparse distribution likely reflects the presence of plus-ends at staggered locations in axons and dendrites (26). We do not know if kinetochore proteins are present at all plus-ends or only some, or principally at stable or growing plus-ends. The copies of MIS12 that were near EB1 may represent only a fraction of the MIS12 in neurons. Although the presence of kinetochore components close to microtubule plus-ends supports a role for the kinetochore in microtubule stabilization, it does not exclude other potential functions – whether in axons, dendrites, or cell bodies. Indeed, our data indicated that kinetochore complexes also remained in neuronal nuclei. Does the kinetochore retain a function at the centromere or elsewhere on neuronal chromosomes?

It is likely that kinetochore functions in neurites are similar to their mitotic functions. During mitosis the centromeric kinetochores catch and stabilize the plus-ends of microtubules prior to chromosome segregation. Several lines of data indicate that the kinetochore similarly serves directly to stabilize microtubules in postmitotic neurons. In *Drosophila* sensory dendrites, knockdown of mis12, ndc80, or spc105R increased the number of EB1-GFP comets, a marker of growing dynamics microtubules (Figure S6). Similarly in mouse dendrites, Ndc80 and Dsn1 deletion increased EB3-tdTomato comets, another marker of growing dynamic microtubules (Figure 5). Because overall tubulin staining was not altered, this increase is likely to reflect destabilization of microtubules rather than a large increase in microtubule number. Similar results in *Drosophila* have been observed by Hertzler et al (7) , although Cheerambathur et al. had not detected such a change in *C. elegans* neurons (3). The proximity of MIS12-GFP to plus-ends (Figure 4E and F) is consistent with a stabilizing role of the KMN complex for microtubule growing ends. Moreover, the influence of Ndc80 mutations on the speed of plus-end growth in both mouse (Figure S8E) and *C. elegans* neurons (Figure 6D), strongly suggests that it is normally present on growing plus ends. In *C. elegans*, where the shrinkage phase could also be visualized, the rate of shortening was not dependent on Ndc80. Thus, it is possible that Ndc80 is released, along with other plus-end binding proteins at the end of the growth phase, and indeed its release may contribute to the destabilization that initiates microtubule shortening.

Altered microtubule stability was clearly linked to the change in dendritic spine formation upon loss of Ndc80 or Dsn1. The spine phenotype caused by Ndc80 deletion could be rescued by re-expression of Ndc80, but only if its microtubule-binding site was preserved (Figure 2E-2I). Even a 3 amino acid change that diminished the affinity of NDC80 for microtubules prevented rescue (Figure 2J-2N). Similarly, microtubule plus-ends invaded spines at a higher rate upon deletion of Ndc80, and this phenotype could be rescued by wildtype Ndc80 but not by Ndc80 lacking its microtubule-binding region (Figure 7). The correlation between microtubule invasions and plus-end dynamics suggests that dynamic microtubules have an intrinsic propensity to enter spines and stabilize them. A similar correlation was reported when disruption of the tubulin detyrosination-tyrosination cycle caused abnormal microtubule dynamics, reduced the number of microtubules entering dendritic spines, and caused significant synapse loss (14).

Although dendritic spine formation mainly depends on the actin cytoskeleton, the importance of microtubules in the process is increasingly appreciated (29, 30). Spine invasions have been known to correlate with NMDA receptor-mediated calcium influx and structural expansion associated with synaptic strengthening (16, 31). Microtubules may help transport specific cargo into or out of the spines; at least one motor-cargo pair has been shown to follow such a scheme (18). However, it has remained unclear to what extent microtubule entries causally contribute to spine structural plasticity. Moreover, while there is evidence that local, activity-dependent accumulation of actin filaments contributes to steering microtubules into spines (32), specific molecular regulators of spine invasions have not been identified. Our results indicate that microtubule entries do induce spine formation and/or maintenance and do so under the unexpected control of Ndc80 and the KMN complex through their modulation of microtubule plus-end behavior.

Kinetochore proteins join a growing list of mitosis-associated proteins that have also been implicated in postmitotic neuronal development. Spindly, a protein that recruits dynein to kinetochores in mitosis, is required for dynein-dependent microtubule sorting in axons of *Drosophila* neurons (33) and three mitotic motors, kinesins 6, 12, and 14a are enriched in cell bodies and dendrites of neurons and influence dendritic and axonal growth and morphology (34-40). Thus, our present study contributes to a growing appreciation that regulators of spindle function are repurposed to assist in the complex organization and dynamics of neuronal microtubules. It is intriguing to consider that the microtubule-stabilizing and kinetochore-anchoring activity of Ndc80 is finely controlled during mitosis by partner proteins, phosphorylation state, and mechanical tension applied to the target microtubule. These regulatory pathways may also prove to be crucial in sculpting neuronal development and synapse formation.

## Materials and Methods

### Contact for reagent and resource sharing

Further information and requests for resources and reagents should be directed to and will be fulfilled by the lead contact, Thomas Schwarz (Thomas.Schwarz@childrens.harvard.edu).

### Experimental models and subject details

#### *Drosophila* stocks

*ppk*-*GAL4* and *UAS-EB1:GFP* were obtained from the Bloomington Drosophila Stock Center and the RNAi *Drosophila* stocks (supplemental table S1) from the Vienna Drosophila Resource Center (VDRC). Wild type refers to *w^1118^*. All the fly stocks were maintained on standard cornmeal food at 25°C. Larval sexes were not determined.

#### *C. elegans* strains

*C. elegans* strains were grown on NGM (Nematode Growth Media) plates at 20°C using OP50 *E. coli* as a food source.

#### Mice

All experimental procedures were performed in compliance with animal protocols approved by the IACUC at Boston Children’s Hospital and Harvard University. Male and female mice were used in this study at ratios dependent on litters available and with equal distributions across experiments conducted extemporaneously.

#### HEK293 cells and culture condition

HEK293T Cells from ATCC were cultured in DMEM (ThermoFisher Scientific) supplemented with L-glutamine, penicillin/streptomycin (ThermoFisher Scientific), and 10% FBS (Atlanta Biologicals).

#### iPSC-derived human neurons and culture conditions

For induction of cortical neurons, the BR33 iPSC line was used (41). Stem cells were plated in Matrigel-coated (Corning, 354230) plates and maintained in StemFlex media (ThermoFisher Scientific, A3349401), dissociated to single cells and passaged about once a week by StemPro^™^ Accutase^™^ Cell Dissociation Reagent (Thermofisher Scientific, A1110501) before differentiation.

Cortical neurons were differentiated according to a protocol published by (42) with minor modifications as described in the detailed method. Neurons were cultured with neurobasal medium (NBM).

#### hippocampal neuron culture

Hippocampal neurons were cultured as in (6, 43). Specifically, hippocampal neurons were obtained from E18 mixed-sex mouse embryonic brains, plated on 24 well glass bottom plates (Cellvis, cat# P24-1.5H-N) coated with 20 μg/mL poly-L-Lysine (Sigma-Aldrich, P2636) and 3.5 μg/mL laminin (ThermoFisher Scientific, 23017015). The neurons were maintained in Neurobasal medium (ThermoFisher Scientific, 21103049) supplemented with B27 (ThermoFisher Scientific, 17504044), L-glutamine (ThermoFisher Scientific, 25030081), and penicillin/streptomycin (Sigma, p4333).

## Supplementary Material

Video S1

Video S2

Video S3

Video S4

supplementary material

## Significance Statement

Dendritic spines are vital structures for neuronal communication, and dysregulation of dendritic spines occurs in many neurological and neuropsychiatric disorders. Here we describe a mechanism by which proteins of the kinetochore complex control dendritic spine formation. Kinetochore proteins are crucial for chromosome mechanics during mitosis but have only recently been demonstrated to function in postmitotic neurodevelopment. The absence of kinetochore proteins increased microtubule plus-end dynamics and microtubule invasions into dendritic spines. The altered microtubule behavior, mediated by the microtubule-binding domain of NDC80, correlates with increased dendritic spines both *in vitro* and *in vivo*. During mitosis, the kinetochore undergoes extensive regulation to coordinate chromosome movements; the present study raises the possibility that similar regulation will control the extent of synapse formation.
