# Supplementary material for: Kinetochore proteins control microtubule dynamics in postmitotic neurons to regulate the formation of dendritic spines
Source: Proc Natl Acad Sci U S A. Author manuscript; Available in PMC 2026 Apr 30. (PMC13132258; doi:10.1073/pnas.2520684123)
Supplement: supplementary material [file NIHMS2161265-supplement-supplementary_material.pdf]

Supporting Information for

Kinetochore proteins control microtubule dynamics in postmitotic neurons to regulate the formation of dendritic spines

Guoli Zhao[1], Aditi Sharma[2#], Jing Tang[1#], Martina Aleman[2#], Xing Liang[3#], Lauren Miner[1], Jingqi Qi[1], Wangchu Xiang[1], Feng Tian[4], Yves Goldberg[2], Zhigang He[1], Kang Shen[3,5], Leticia Peris[2*], Thomas Schwarz[1*]

1. F.M. Kirby Neurobiology Center, Boston Children's Hospital, Boston, MA 02115, USA
2. Univ. Grenoble Alpes, Inserm, U1216, CNRS, Grenoble Institut Neurosciences, 38000 Grenoble, France
3. Department of Biology, Stanford University, Stanford, CA 94305, USA
4. Department of Neurology, Beth Israel Deaconess Medical Center, Harvard Medical School, Boston, 02115, MA, USA.
5. Howard Hughes Medical Institute, Department of Biology, Stanford University, Stanford, CA 94305, USA

# Equal contribution.
* Corresponding authors: Thomas.Schwarz@childrens.harvard.edu and leticia.peris@univ-grenoble-alpes.fr.

This PDF file includes:

Supporting text
Figures S1 to S8
Tables S1
Legends for Movies S1 to S4
SI References

Other supporting materials for this manuscript include the following:

Movies S1 to S4

Supporting Information Text

METHOD DETAILS

Plasmids

pLXV-hSyn-Halo-DSN1, pLXV-hSyn-Halo-NDC80, pLXV-hSyn-Halo-Ndc80-3KE and pLXV-hSyn-Halo-∆MTB-NDC80 were constructed using the Gibson assembly (NEB, E2611) method. The pLXV backbone and coding sequences for Halo tag ,DSN1, human NDC80, human NDC80 with three ammino acids mutations (where the lysine was mutated to glutamic acid at positions 89, 115, 166) and microtubule binding deficient form of human NDC80 (where the lysine was mutated to glutamic acid at positions 89, 115, 166 and amino acids 1-79 amino acids were deleted) were amplified by PCR and the PCR products were assembled by Gibson assembly according to the manufacturer's protocol. For making plasmids, pLXV-hSyn-GFP-NDC80 and pLXV-hSyn-GFP-∆MTB-NDC80, the Halo coding sequence in pLXV-hSyn-Halo-NDC80 and pLXV-hSyn-Halo-∆MTB-NDC80 was replaced with GFP coding sequence by digestion of the plasmids with the restriction enzymes BamH1 and Afe1 and then ligation of a PCR-amplified GFP sequence with T4 DNA ligase ligation. The correct constructs were identified by whole plasmid sequencing. The plasmid AAV: hSyn-Cre-2A-EGFP which deleted the KASH sequence from the original plasmid AAV:ITR-U6-sgRNA(backbone)-hSyn-Cre-2A-EGFP-KASH-WPRE-shortPA-ITR (Addgene, 60231) was made by Q5-site-directed mutagenesis (NEB, Q0552S) method. AAV: hSyn-∆Cre-2A-EGFP plasmid, designed to express Cre lacking the last 221 amino acid, was made by Q5-site-directed mutagenesis using AAV: hSyn-Cre-2A-EGFP as the original plasmid. For constructing plasmid hMis12-HDR-TOPO, the synthesized HDR fragment carried the EGFP coding sequence and sequences homologous to the Mis12 genomic region to either side of the targeted insertion site. This fragment was cloned into the Blunt II-TOPO cloning vector (Thermofisher, 45-024-5) according to the manufacturer's protocol. The hMis12-gRNA-PX459 plasmid was made by annealing the synthesized guide RNA oligos (IDT) with the BbsI digested vector (Addgene, PX459).

The cDNAs encoding LifeAct (aa 1-17 of Saccharomyces cerevisiae ABP140, NP_014882) C-terminally fused to RFP, and human EB3 (NP_001289979) C-terminally fused to YFP were cloned in tandem in pLV-mCherry. During the cloning process, the mCherry sequence was removed and replaced by an IRES (encephalomyocarditis virus internal ribosome entry site) sequence to generate the bicistronic vector pLV-LifeAct-IRES-EB3 for co-expression of LifeAct-RFP and EB3-YFP. PCR amplification and cloning of cDNAs was performed with Phusion DNA polymerase (Thermo Scientific) and In-Fusion HD Cloning kit (Clontech), respectively. The construct was verified by sequencing (Eurofins and Genewiz) and purified with HiPure Plasmid).

AAV Virus production

For Cre expression, we used a plasmid derived from AAV:ITR-U6-sgRNA(backbone)-hSyn-Cre-2A-EGFP-KASH-WPRE-shortPA-ITR (Addgene, 60231) from which the KASH had been removed. A recombination-deficient version of Cre (ΔCre) was used as control (1). These plasmids were used by Boston Children's Hospital Viral Core to prepare retro serotype AAV for transducing spino-cortical neurons in vivo. The titers of all viral preparations were at least 5 x 10^{12} genome copies/ml.

Lentivirus production

pFSW-hSyn-NLS-Cre-mCherry, pFSW-hSyn-NLS-ΔCre-mCherry, pFSW-hSyn-NLS-Cre-GFP, pFSW-hSyn-NLS-ΔCre-GFP, pLXV-hSyn-Halo-DSN1, pLXV-hSyn-Halo-NDC80, pLXV-hSyn-Halo-Ndc80-3KE, pLXV-hSyn-Halo-ΔMTB-NDC80, and pLV-LifeAct-IRES-EB3 plasmids were packaged in lentivirus by co-transfecting HEK293 cells with plasmid psPAX2 (Addgene, 12260) and a VSV-G envelope expressing plasmid (either pMD2.G; Addgene, #12259 or pCMV-VSV-G; Addgene plasmids # 8454). The viral particles for Cre and Ndc80 expression were purified by centrifugation and resuspended in DPBS+0.001% F68 (Thermofisher, Cat#24040032). Viral particles for LifeAct and EB3 expression were collected 48 h after transfection by ultra-centrifugation of filtered culture supernatants prior to aliquoting. All viral particles were flash-freezing in liquid nitrogen and storage at -80°C.

Immunohistochemistry of mouse brain slices

Mice were euthanized with > 5% isoflurane for 5 min and then perfused with PBS and 4% PFA/PBS. Dissected brains were immersed in 4% PFA/PBS for post-fixation overnight at 4 ℃. Brains were washed with PBS for 4 times, transferred to 30% sucrose in PBS at 4 °C and then to OCT embedding medium (Tissue-Tek) and frozen at -20 °C for at least 1 hour. Sections of 40 μm thickness were cut, washed twice in PBS, and permeabilized with 0.5% Triton-X100 (PBST) for 20 minutes. Sections were then washed twice with PBS before blocking with SuperBlock buffer (ThermoFisher, Cat # 37515) for 30 minutes. All washes were 10-15 minutes. Brain slices were incubated with primary antibodies in blocking buffer overnight at 4°C. After 5 washes with PBS, they were incubated with secondary antibodies in blocking buffer for 3 hours at room temperature. After 5 washes with PBS, slices were mounted in Fluoromount-G Anti-Fade mounting medium (SouthernBiotech, cat# 0100-35).

We used chicken anti-GFP (1:1000; Aveslab, GFP-1010) as primary antibody, and Alexa Fluor-488 conjugated antibody (1:400; ThermoFisher Scientific) as secondary antibody. The brain slices were imaged with an LSM700 laser-scanning confocal microscope (Zeiss) and a 63× 1.4 NA objective using separate channels and processed using the Fiji (ImageJ).

Generation of conditional knockout mice

The Dsn1 and Ndc80 conditional knockout mice were generated by the Gene Manipulation Core at Boston Children's Hospital. CRISPR reagents and injection: A cocktail of 0.61pmol/μl each of crRNA and tracrRNA, 100ng/μl Cas9 protein and 10ng/μl ds-Donor DNA were injected into the pronuclei of E0.5 embryos (C57Bl6/Hsd). Surviving post-injection embryos were re-implanted into recipient CD1 pseudo-pregnant females and allowed to develop to term. The resultant pups were genotyped and genome-edited founders identified and mated to establish lines (2).

In detail, we designed and synthesized the homology-directed repair (HDR) template carrying flanking loxP sequences and homology arms for Ndc80 or Dsn1 as diagrammed in Supplemental Figure 1. The HDR template was injected with crRNA, tracrRNA and Cas9 protein into the pronuclei of E0.5 embryos to make the floxed alleles of these genes. For the Dsn1 conditional knockout mice, in addition to inserting the loxP sequences, we also inserted a Halo tag at its N-terminal to aid in localizing the protein (figure 1 A and figure S1A). For both Ndc80 and Dsn1 lines, the presence of the loxP sequences was confirmed by PCR amplification of insertion sites and sequencing. To genotype Dsn1 floxed mice, we amplified the DNA sequence around the insertion site using primers one either side of the insertion site. The expected PCR product size after loxP and Halo insertions in Dsn1 is 1693 bp and for wildtype is 610 bp (Figure 1E lane 1 and lane 3). For genotyping Ndc80 floxed mice, a BamHI cutting site was introduced along with the 5' loxP site (Figure S1B). We amplified the DNA sequence around the insertion site by PCR and the PCR product was digested by BamHI to give two bands (805 bp and 493 bp).

Generation of the C. elegans NDC-80::ZFGFP knock-in strain

The NDC-80::ZFGFP knock in worm was generated by CRISPR/Cas9 editing (3).

***C. elegans* synchronization and staging**

C. elegans embryos were collected using standard bleaching technique. The embryos were then transferred to unseeded NGM plates and incubated at 20 degree overnight to obtain synchronized L1 stage larvae worms. The L1-arrested population was transferred to OP50-seeded NGM plates and grown for 28-30h before imaging MT dynamics in the posterior primary dendrite of PVD.

CRISPR insertion of GFP tag into iPSC Mis12

To insert an EGFP tag at the N-terminal of MIS12 in the BR33 iPSC line, we designed a guide RNA around the start codon of Mis12. The repair template (HDR) was synthesized to contain 500 bp of homology sequences either side of the insertion site and the EGFP coding sequence and this DNA was cloned into the Blunt II-TOPO cloning vector (Thermofisher, 45-024-5). The guide RNA was synthesized by IDT and cloned into a construct that expresses Cas9 (Addgene, PX459).

The iPSC cells were maintained in StemFlex media (ThermoFisher, A3349401) and fed every other day. For the CRISPR knock-in, a 6-well dish was coated with growth factor-reduced Matrigel (Matrigel-GFR #354230, Corning). Prior to nucleofection, cells were grown to confluency in a flask and then trypsinized with 3 mL StemPro Accutase (A1110501, Thermofisher) for 10

minutes which was then quenched by adding 7ML of mTeSR-1 (STEMCELL Technologies, #85850). Cells were transferred to a 50 mL conical tube and then to Eppendorf tubes such that each tube contained an aliquot portion of 2 million cells. Cells were pelleted at 500 rcf for 5 min prior to resuspension in 100µl nucleofector mix (18µl Nuleofector supplement and 82 µl nucleofector solution from the Human Stem Cell Nucleofector Kit 2 (Lonza, VPH-5022)) with 3ug each of hMis12-HDR-TOPO and hMis12-gRNA-PX459 plasmid. Cells were rapidly transferred to a cuvette and electroporated under program B20 after which 1mL RPMI media (A1049101, Gibco) containing ROCK inhibitor (Y27632, StemCell Technologies) was immediately added. After 20 min incubation at 37C, 2 mL of mTesR-1+ROCK inhibitor were added and cells were gently pipetted into 6-well plates containing mTeSR-1+ROCK inhibitor. On day 2, media was changed to 3 mL StemFlex and replaced on day 3, with 3 mL fresh StemFlex with puromycin (1ug/mL). On day 4, medium was removed and cells were gently washed with PBS, before fresh StemFlex (2.5 mL) was added. On day 5, medium was again removed, cells were washed twice with PBS and 1 mL accutase was added for 7 min incubation at 37℃. Cells were pelleted and resuspended in 2 mL StemFlex with ROCK inhibitor. The cell suspension was moved to a new tube, centrifuged at 500 rcf for 5 mins, the medium was removed, and cells were-suspend with 500 uL PBS with Rock inhibitor (1:1000). GFP-positive cells were isolated by FACS sorting and plated at one cell per well into 96 well plates. Positive clones were identified by PCR and sequencing confirmed the correct insertion.

For analyzing Mis12 expression in iPSCs and iNeurons, 1 million BR33 or EGFP-Mis12 iPSCs and D10 iNeurons were collected and lysed in RIPA buffer (Boston BioProducts, BP-115) with proteinase inhibitor (Millipore Sigma, 11836170001). The samples were blotted with anti-human MIS12 complex antibody (4) the mouse anti-GAPDH (Millipore Sigma, CB1001) antibody as a loading control.

Differentiation and Immunostaining of iNeurons

BR33 and EGFP-MIS12 iPSCs were differentiated into cortical neurons according to a published protocol (5). To transduce iPSCs with NGN2-expressing virus, 12 well plates were coated with growth-factor-reduced Matrigel (#354230 Corning) and iPSCs were plated with 380k cells per well (~100k cells/cm^2) in mTeSR media plus ROCK inhibitor (10 uM) on day 0. Cells were infected with pTet-O-NGN2-puro and Fudelta GW-rtTA lentivirus on day 1. On day 2, when cells were confluent, each well of the 12 well plate was used to seed one 10 cm plate. On day 3-7, cells were fed daily with StemFlex and, when confluent, were split for maintenance and expansion. For generating iNeurons, the transduced iPSCs were plated at 200k cells/cm^2 with StemFlex media plus ROCK inhibitor (10 uM) in 10cm plates. The next day (D1), when cells were at least 75% confluent, cells were fed with KSR media with doxycycline (2ug/mL) to induce Ngn2 expression. On day 2 (D2), cells were fed with 1:1 ratio of KSR:N2B media with puromycin (5 ug/mL) and doxycycline (2ug/mL) to select for transduced cells. Cells were fed with N2B media

with B27 (1:100), puromycin (5ug/mL), and doxycycline (2ug/mL) on day 3 (D3). On day 4 (D4), the 10cm plates were washed with 3 mL PBS and add 3 mL/plate of diluted accutase (Gibco, A11105) solution (1:3 accutase:PBS with ROCK inhibitor). Cells were incubated for 3-5 minutes until they had lifted off the plate. Cells were then collected, counted and pelleted before plating at 37.5k cells/cm^2 in plating media (NBM+B27 (1:50) with ROCK inhibitor (10 uM), BDNF/GDNF/CNTF (10 ng/mL), puromycin (5ug/mL), and doxycycline (2 ug/mL). On day 5 (D5), all the media was replaced with the same plating media, but without ROCK inhibitor. Cells were fed every 3 days with half-media changes using plating media without ROCK inhibitor.

For immunostaining, D14 iNeurons were fixed with 4% PFA in PBS for 12 minutes, washed once with PBS, and permeabilized with 0.5% PBST (triton x-100) for 5 minutes. After three washes with PBS, they were placed in Super Block buffer (ThermoFisher, 37515) for 30 minutes prior to incubation with rabbit anti-GFP (Life technology, A11122) and mouse anti-Tuj1 (Biolegend, 801202) antibodies overnight at 4C. The next day, after 3 10-minutes washes with PBS the cells were incubated with Hoechst (Thermofisher, 62249), phalloidin-Atto 647N (Millipore Sigma, 65906), goat anti-rabbit-alexa-488 (Thermofisher, A11034) and goat-anti-mouse-alexa-568 (Thermofisher, A11031) for 3 hours at room temperature. After 3 10-minute washes with PBS, samples were mounted with Fluoromount-G® Anti-Fade mounting medium (SouthernBiotech, 0100-35).

Analysis of hippocampal neurons

DIV 10 or DIV14 hippocampal neurons were infected with lentivirus pFSW-hSyn-ΔCre-mCherry (control) or pFSW-hSyn-Cre-mCherry and, when indicated, also with pLXV-hSyn-Halo-Ndc80, pLXV-hSyn-Halo-ΔMTB-Ndc80, or pLXV-hSyn-Halo-Dsn1. On DIV12 or DIV16 neurons were transfected using NeuroMag transfection reagent (OZBioscieces, NM50500) with FUmGW (Addgene, 22479) to express membrane-bound GFP. On DIV 15 or DIV19, cells were fixed for 10 min with 4% paraformaldehyde, 4% sucrose in phosphate-buffered saline at room temperature. After 3 10-minute washes with PBS and blocking with Superblock buffer for 30 minutes, cells were immunostained in Superblock blocking buffer (ThermoFisher, 37515) overnight at 4℃ with rabbit anti- GFP antibody (ThermoFisher Scientific, A11122) or chicken anti-GFP (Aveslabs, GFP-1020) at 1: 1000, rabbit anti-Synapsin (Abcam, ab254349) at 1:500, and mouse anti-PSD95 (Abcam, ab192757) at 1:500 and subsequently incubated with goat anti-rabbit Alexa-488 or goat anti-chicken Alexa-488, goat anti-mouse Alexa-568 and goat anti-rabbit Alexa-647 secondary antibody (1:500 in Superblock) for 3 hours at room temperature. The stained samples were mounted with cover slips using Fluoromount-G® Anti-Fade mounting medium (SouthernBiotech, 0100-35). Z-stack images were acquired for quantification of dendritic protrusions using a Zeiss LSM700 confocal microscope (Zeiss, Thornwood, NY). All transfected neurons with a pyramidal morphology that expressed both GFP and mCherry (which marked the Cre or inactive Cre expressing cells) were imaged in an unbiased manner. To quantify dendritic protrusions, neurons

were selected based on GFP staining at a low magnification, at which protrusions were not visible, so as not to bias the selection. Dendritic segments that were 20-30 μm from the cell body were then examined at higher magnification. For each phenotype, at least 14 neurons from two independent experiments were examined. Optical sections of dendrites were acquired with a 63X, 1.4 NA oil immersion objective with 0.5 μm intervals and 1024x1024 pixel resolution and averaged twice. Dendritic protrusions were then analyzed manually by using Fiji ImageJ software (available at https://fiji.sc/).

Live imaging of EB3 or EB1 comets for Figures 6, and S6

Hippocampal neurons were infected on DIV6 with lentivirus pFSW-hSyn-ΔCre-GFP or pFSW-hSyn-Cre-GFP and, when indicated, also with pLXV-hSyn-Halo-Ndc80, pLXV-hSyn-Halo-ΔMTB-Ndc80 or pLXV-hSyn-Halo-Dsn1. Neurons were transfected on DIV8 with EB3-tdTomato (Addgene, 50708) using Lipofectamine 2000 (Life Technologies, 11668-019) and imaged on DIV11 on a Nikon Ti-Eclipse inverted microscope with a 60X, 1.4 NA oil immersion objective. The neuron chamber was maintained at 37 ℃ and supplied with 5% CO_2. The movies were taken at 1 Hz for 3 minutes and analyzed by Kymograph.

Late third-instar Drosophila larvae were dissected in calcium-free HL3 saline (6) (in mM, 70 NaCl, 5 KCl, 20 MgCl2, 10 NaHCO3, 5 trehalose, 5 HEPES, 115 sucrose; pH 7.3) while keeping the central nervous system intact. Dendritic branches of type IV sensory neurons were imaged using a Zeiss LSM710 microscope with 40x water-immersion lens at 1 frame each second for 3 minutes.

Imaging for microtubule in C. elegans

Imaging of microtubule dynamic was performed on an inverted Zeiss Axio Observer Z1 microscope equipped with a Yokogawa spinning disk, QuantEM:512SC Hamamatsu camera (set to 600 EM Gain), a Plan-Apochromat 100x/1.4 NA objective (Zeiss), 488 nm lasers, and controlled by MetaMorph Microscopy software. Movies were acquired using the following parameters: 30% laser power, 200ms exposure time and 500 frames captured per movie.

Proximity ligation assay (PLA)

Proximity ligation assay for MIS12 localization in neurons followed the manufacturer's protocol. iPSC-derived neurons (iN) on day 6 to day 20 were fixed in 4% PFA in PBS for 12 minutes. For co-staining of MIS12 with EB1, the neurons were fixed in cold methanol containing 1 mM EGTA at -20℃ for 6 minutes, then fixed in 4% PFA, 4% sucrose PBS for 6 minutes at room temperature. For PLA experiments not involving EB1, the methanol step was omitted. After washing once with PBS, samples were permeabilized with 0.5% PBST (triton X-100) for 5 minutes, washed three times with PBS, and then placed in blocking solution (supplied in the PLA kit, Sigma, UDO92101-1KT). Samples were incubated with primary antibodies diluted in antibody diluent. The primary antibodies used were rabbit anti-GFP, mouse anti-Flag, mouse anti-EB1 and rabbit anti-Ndc80 complex. After primary antibody incubation, the samples were washed twice for

5 minutes in 1x Wash Buffer A at room temperature and then incubated with PLA probe solution at 37℃ for 1 hour. Samples were washed twice for 5 minutes in 1x Wash Buffer A at room temperature and incubated with ligation solution for 30 minutes at 37℃. After washing twice for 5 minutes in 1x Wash Buffer A at room temperature, samples were incubated with amplification solution for 100 minutes at 37℃. The final washes were performed twice for10 minutes in 1x Wash Buffer B at room temperature, and then in 0.01x Wash Buffer B for 1 minute prior to mounting.

Proximity ligation assay for Ndc80 and ∆MTB-Ndc80 colocalization with EB1 was done in cultured ndc80$^{fl/fl}$ mouse cortical neurons. The lentiviruses expressing Cre-mCherry, Ndc80 or ∆MTB-Ndc80 under control of the synapsin promoter were added to the medium for DIV3 neurons. 4 days after infection, the neurons were fixed and the PLA assay was done according to the same protocol as described for iNeurons. The primary antibodies used were rabbit anti-GFP and mouse anti-EB1. Rat anti-tyrosinated tubulin was used to label the neurites and Hoechst dye to label the nucleus.

Western blot for detecting the expression level of GFP-NDC80

Primary mouse cortical neurons were seeded at a density of $1x10^6$ cells per well of 6 well plates and 1 well was transduced at DIV3 with a lentiviral vector expressing either wild-type Ndc80 or the microtubule-binding mutant (∆MTB-Ndc80) under the control of a synapsin promoter. Four days post-infection, neurons were harvested and lysed in 120 µL/well of RIPA buffer supplemented with a 1:100 dilution of protease inhibitors. Lysates were incubated on ice for 30 minutes, followed by centrifugation at 12,000 rpm for 10 minutes at 4°C. The resulting supernatants were collected, combined with 40 µL of 4× Laemmli sample buffer, and denatured by boiling for 10 minutes. Following equilibration to room temperature, 20 µL of each sample was resolved via 12% SDS-PAGE and analyzed by Western blotting. Target proteins were detected using rabbit anti-GFP, mouse anti-GAPDH, and rabbit anti-Tuj1 primary antibodies.

Live imaging of EB3 comets for Figure7 and Supplemental Figure 8

Hippocampal neurons derived from Ndc80$^{flox/flox}$ embryos were thawed from a stock that had been frozen and shipped in CryoThrive (Transnetyx) according to the manufacturer’s protocol and were then grown in poly-lysine-coated glass-bottom dishes that had been fitted with an insert that creates four wells atop the culture area (Ibidi). The neurons in all four wells of a dish were infected at 7 DIV with the LifeAct-RFP-EB3-YFP virus (m.o.i. =5). Then at 11 DIV three of the wells received the mCherry-Cre virus, while the remaining well was infected with the control mCherry virus. Finally at 12 DIV, one of the three Cre-expressing cultures was rescued by infection with wild-type, Halo-tagged Ndc80, and a second one received the microtubule binding deficient form, ΔMTB-Ndc80. Imaging of live neurons was performed using a 63×/1.46 N.A. oil objective (Zeiss) mounted on an inverted microscope (Axio Observer, Zeiss) coupled to a spinning-disk confocal system (CSU-W1-T3, Yokogawa) via a structured illumination module

(Live SR, GATACA Systems, France) allowing fast modulation of illumination patterns for high-speed super-resolution. Emitted light was detected with an electron-multiplying charge-coupled device (CCD) camera (ProEM+1024, Princeton Instrument). The whole system was controlled by Metamorph software (Molecular Systems). The microscope was housed in a box kept at 37°C to maintain thermal equilibrium with the sample. At 18 DIV, the dishes were transferred to the microscope stage in an on-stage incubator (LaCon GmbH, Germany) supplied with humidified 5% CO2 in air at 37°C. Fluorescence was excited by combined beams of 488 nm and 561 nm laser light (5 mW/mm^2 at objective pupil for each wavelength) delivered by two diode lasers (GATACA Systems, France). Using image-splitting dichroic mirrors included in the confocal spinning unit, YFP and RFP were simultaneously imaged on separate halves of the CCD detector in split-view mode. Stacks of optical sections (4 planes per stack, 0.7 µm step size, 200 ms exposure per frame) were acquired every 5 s in 5 min-long video sequences. Super-resolved images of all frames of a time series were then generated by mathematical reconstruction of high-spatial frequency features (Gataca systems software). The resulting image sequences were analyzed with ImageJ.

For each stack of a video sequence, a maximum intensity projection was generated in both channels. To locate transient entries of microtubules into spines, the projections from all time points in a sequence were superposed in a final maximum intensity projection. Spine invasions were then manually spotted by searching for small cilium-like shapes extending out of the YFP-labeled shafts and into RFP-labeled protrusions. The percentage of the spines invaded was calculated as the number of spines invaded by microtubules during a 5 min film divided by the total number of spines of the same dendrite in the imaged field. To analyze microtubule dynamics, kymographs were generated by drawing a region of interest on the middle part of the dendrite.

Parameters describing microtubule dynamics were measured using a home-made ImageJ plugin and defined as follows: comet density: number of full tracks per surface of the dendrite (µm²) per min; catastrophe frequency: number of full tracks / total duration of growth; nucleation/rescue frequency: number of tracks that originate in the kymograph per surface of the dendrite (µm²) per min; growth length: comet movement length in µm; comet lifetime: duration of growth; growth rate: growth length / comet lifetime.

QUANTIFICATION AND STATISTICAL ANALYSIS

Statistical analysis

Statistical analysis of data was done using GraphPad Prism software. P values were derived using a student’s t-test with Welch-correction or One-way ANOVA with Tukey's multiple comparisons test as indicated. Graphs were plotted in GraphPad Prism using the scatter dot plot

functions showing the mean and standard error of the mean (SEM). P values for each experiment are indicated in the figures or figure legends.

The data on dendritic spines and microtubule dynamics, both in vitro and in vivo, have two potential sources of variability – from neuron to neuron and from culture to culture (or animal to animal). Because the neuron-to-neuron variability was very large and the variability from culture to culture insignificant by comparison, we treated each neuron as an independent event so as not to minimize the true extent of the observed variance.

To measure MT dynamic length and dynamic speed in C. elegans: Kymograph was made using the Reslice function in ImageJ, and the length and angle of the TBA-1 growing or shrinking lines were measured as lp and α, as the scale for distance is 0.109μm/pixel, the length was calculated using the formula: $l=0.109*lp*\cos\alpha$ and the speed was calculated by the formula: $speed(\mu m/s)=0.109/t*\cot\alpha$, t is the time interval. For MT length measurement, we included only those events in the kymographs that displayed the complete polymerization/depolymerization cycle. For MT speed and dynamic frequency quantification, all events observed were counted.

To plot the MT growth speeds at different time points, events between the growth start site and shrinkage start site were divided into 10 equal segments by time, marked by 11 reference points, and the xy coordinates of these points were obtained using imageJ. The growth speed was then calculated using the formula: $speed=0.109*\Delta x/t*\Delta y$, Δx is the pixel distance between adjacent points while Δy is the pixel time difference between adjacent points.

Supplemental table S1. Key resources table. The table contains all crucial reagents used in this study.

REAGENT or RESOURCE	SOURCE	IDENTIFIER
**Antibodies**		
Chicken anti-GFP	Aveslabs	GFP-1010<br>RRID:AB_10000240
Rabbit anti-GFP	Life technology	A-11122<br>RRID: AB_221569
Rabbit anti-Tubulin β-3 (TUBB3)	BioLegend	802001
Mouse anti-Tubulin β-3 (TUBB3)	BioLegend	801202
Rat anti-tyrosinated tubulin	abcam	Ab6160<br>RRID:AB_305328
Mouse anti-EB1	BD Bioscience	610534<br>RRID: AB_397891
Rat anti-tyrosinated tubulin	Abcam	Ab6160<br>RRID: AB_305328
Mouse anti-PSD95	Thermofisher	MA1-046<br>RRID: AB_2092361
Mouse anti-PSD95	Abcam	Ab192757<br>RRID: AB_2750929
Rabbit anti-Synapsin1	Abcam	ab254349<br>RRID: AB_2920663
Rabbit anti-human Ndc80 complex	Iain Cheeseman lab	N/A
Rabbit anti-human Mis12 complex	Iain Cheeseman lab	N/A
Mouse anti-HEC1/NDC80	Abcam	Ab3613<br>RRID:AB_303949
rabbit anti-TUBA1A	Millipore Sigma	SAB4500087<br>RRID:AB_10743646
Mouse anti-FLAG	Millipore Sigma	F1804<br>RRID:AB_262044
Rabbit anti-GAPDH	Santa Cruz Biotechnology	Cat# SC-25778
Mouse anti-GAPDH	MilliporeSigma	CB1001
Mouse anti-GAPDH (6C5)	Millipore Sigma	CB1001<br>RRID:AB_2107426
HaloTag® TMR Ligand	Promega	G8251
Goat anti-Chicken IgY (H+L) Secondary Antibody, Alexa Fluor™ 488	Thermofisher	A-11039
Phalloidin–Atto 647N	Millipore Sigma	65906
Goat anti-Rabbit IgG (H+L) Highly Cross-Adsorbed Secondary Antibody, Alexa Fluor™ 568	Thermofisher	A-11036

Alexa Fluor® 568 Goat Anti-Mouse IgG (H+L) Antibody, highly cross-adsorbed	Thermofisher	A-11031
Goat anti-Rabbit IgG (H+L) Highly Cross-Adsorbed Secondary Antibody, Alexa Fluor™ 488	Thermofisher	A-11034
**Critical Commercial Assays**		
Proximity ligation assay (PLA)	Millipore Sigma	DUO92101-1KT
**Experimental Models: Cell Lines**		
HEK293T	ATCC	CRL-3216<br>RRID: CVCL_0063
BR33 (iPSC for cortical neuron)	From Matthew J. LaVoie lab	
EGFP-Mis12	This study	
**Experimental Models: Organisms/Strains**		
w1118; P{GD14729}v29337<br>*ndc80 RNAi*	Vienna Drosophila RNAi Center (VDRC)	29337
P{KK106153}VIE-260B<br>*Spc105R RNAi*	Vienna Drosophila RNAi Center (VDRC)	109322
P{KK113187}VIE-260B<br>*Mis12 RNAi*	Vienna Drosophila RNAi Center (VDRC)	105052
w[*]; P{w[+mC]=ppk-GAL4.G}3	Bloomington Drosophila Stock Center	32079
Dmel\P{UAS-EB1-GFP}3	Bloomington Drosophila Stock Center	35512
C. elegans strain: *ndc-80*(*wy1954*[ndc-80::*zf1*::*gfp*]);*tba-1*(*ok1135*);wyIs813[P*unc-86*::GFP::TBA-1]; *zif-1*(*gk117*);*wyIs1017*[P*unc-86*::ZIF-1 P*odr-1*::*gfp*]	This study	TV29235
C.elegans strain: tba-1(*ok1135*);wyIs813[P*unc-86*::GFP::TBA-1];*zif-1*(*gk117*);*wyIs1017*[P*unc-86*::ZIF-1 P*odr-1*::*gfp*];	This study	TV30933
**Oligonucleotides**		
Dsn1-5loxP-1	AAGACCTTCCACGGAACTGG	gRNA for 5' loxp insertion of Dsn1
Dsn1-Halo-1	ACTGACGTCATCCTGGATGA	gRNA for Halo tag insertion of Dsn1
Dsn1-3loxP-1	CTTTGGAATCACACGTGTGT	gRNA for 3' loxp insertion of Dsn1
Dsn1-5loxP-F	TAGCCCCCAACGAGGATCCATA	Primer for screening for 5'loxP of Dsn1

Dsn1-g5P-R1	CCAAGCTGGAGTCT GTGTGAT	Primer for screening for 5'loxP of Dsn1
Dsn1-g3P-F1	TATGAACGCAGCATC CCACC	Primer for screening for 3'loxP of Dsn1
Dsn1-3loxP-R	GTGTGACGCGTAGAT AACTTCGTA	Primer for screening for 3'loxP of Dsn1
Dsn1-halo-up	CTATTTCCCTGGTGA TGGAAACC	Primer for screening for Halo tag insertion of Dsn1
Dsn1-halo-down	CTGCCAAAGAGAAGT GACTGCG	Primer for screening for Halo tag insertion of Dsn1
Ndc80-5loxP-1	GCGATCTGTCTAACC CTCTG	gRNA1 for 5' loxp insertion of Ndc80
Ndc80-3loxP-1	TCCCAGTAGTACTGG ATTAG	gRNA1 for 3' loxp insertion of Ndc80
Ndc80-5loxP-F	GCTTCCACAGACGA GGATCC	Primer of screening for 5'loxP knock-ins
Ndc80-g5P-R1	GTTTTCGTGTGCAGT CGTGC	Primer of screening for 5'loxP knock-ins
Ndc80-g3P-F1	GTGACCACACCGAG CTATGTA	Primer of screening for 3'loxP knock-ins
Ndc80-3loxP-R	AGTAGTACTGGATTG ACGCGTAG	Primer of screening for 3'loxP knock-ins
Ndc80-Ex2-F	AGGGGAGGCACTTA CGTTTG	Genotyping primer for 5' loxp of Ndc80
Ndc80-g5P-R1	GTTTTCGTGTGCAGT CGTGC	Genotyping primer for 5' loxp of Ndc80
Ndc80-g3P-F1	GTGACCACACCGAG CTATGTA	Genotyping primer for 3' loxp of Ndc80
Ndc80-mid-R	GCCGAGTGCATAGT GTCTGA	Genotyping primer for 3' loxp of Ndc80
Mis12-mEGFP-gRNA1	AAAATAAGCCAAGAT GTCTG	gRNA for knockin GFP into Mis12 N terminal of iPSC
hMis12-KI-R	AAAACAGTTGCTCCA TTTTGCTAAAAAGG	Primer for screening GFP tag insertion at Mis12 N terminal in iPSC
hMis12-KI-F	GTCACAGGTAAGGG AAGTGAACAACG	Primer for screening GFP tag insertion at Mis12 N terminal in iPSC
NDC-80-ZF1-GFP HA-F	AAAAGATAGGATTCC ACGTGCAATGTGCTG GCATTGAAAAGGCG GTTCTTGGATCAGGT TCTGGATCTGGTACC ATGCCGACAGAATAC AAAACGCG	Repair homology arm for ZF1-GFP knock in at NDC-80 C terminus in *C.elegans*
NDC-80-ZF1-GFP HA-R	tttaatgaaagctagtattattca cagaatgattatgccaaagca gaCTATTTGTATAGTT CATCCATGCCATGTG	Repair homology arm for ZF1-GFP knock in at NDC-80 C terminus in *C.elegans*
NDC-80-ZF1-GFP gRNA	tcacagaatgattatgccaa	gRNA for knockin ZF1-GFP into NDC-80 C terminal of *C.elegans*
**Recombinant DNA**		

pLXV-hSyn-Halo-Ndc80	This study	N/A
pLXV-hSyn-Halo-ΔMTB-Ndc80	This study	N/A
pLXV-hSyn-GFP-Ndc80	This study	N/A
pLXV-hSyn-GFP-ΔMTB-Ndc80	This study	N/A
pLXV-hSyn-Halo-Ndc80-3KE	This study	N/A
pLXV-hSyn-Halo-Dsn1	This study	N/A
FUmGW	addgene	Cat# 22479
EB3-tdTomato	addgene	Cat# 50708
GFP-MT+TIP	Casper Hoogenraad lab	
AAV:ITR-U6-sgRNA(backbone)-hSyn-Cre-2A-EGFP-KASH-WPRE-shortPA-ITR	addgene	Cat# 60231
AAV: hSyn-Cre-2A-EGFP	This study	
AAV: hSyn-ΔCre-2A-EGFP	This study	
pFSW-hSyn-NLS-Cre-GFP	Michael Greenberg lab	
pFSW-hSyn-NLS-ΔCre-GFP	Michael Greenberg lab	
pFSW-hSyn-NLS-Cre-mCherry	Michael Greenberg lab	
pFSW-hSyn-NLS-ΔCre-mCherry	Michael Greenberg lab	
hMis12-HDR-TOPO	This study	N/A
hMis12-gRNA-PX459	This study	N/A
TRIM46-mCherry	Casper C. Hoogenraad lab	(7)
pLV-LifeAct-RFP-IRES-EB3-YFP		
**Software and Algorithms**		
Fiji	Schindelin, J.; Arganda-Carreras, I. & Frise, E. et al. (2012)	https://fiji.sc/
Adobe illustrator	adobe	https://www.adobe.com/products/illustrator.html
GraphPad Prism	GraphPad Software	https://www.graphpad.com/scientific-software/prism/
Other		https://www.zeiss.com/microscopy/int/confocal.html
LSM700 laser-scanning confocal microscope	Zeiss	https://www.micro-shop.zeiss.com/?p=us&f=o&a=v&m=s&id=440762-9904-000
63× 1.4 NA objective	Zeiss	https://www.leica-microsystems.com/products/confocal-microscopes/p/leica-tcs-sp8/
TSC SP8 laser scanning confocal microscope	Leica	

Supplemental Figures and Legends

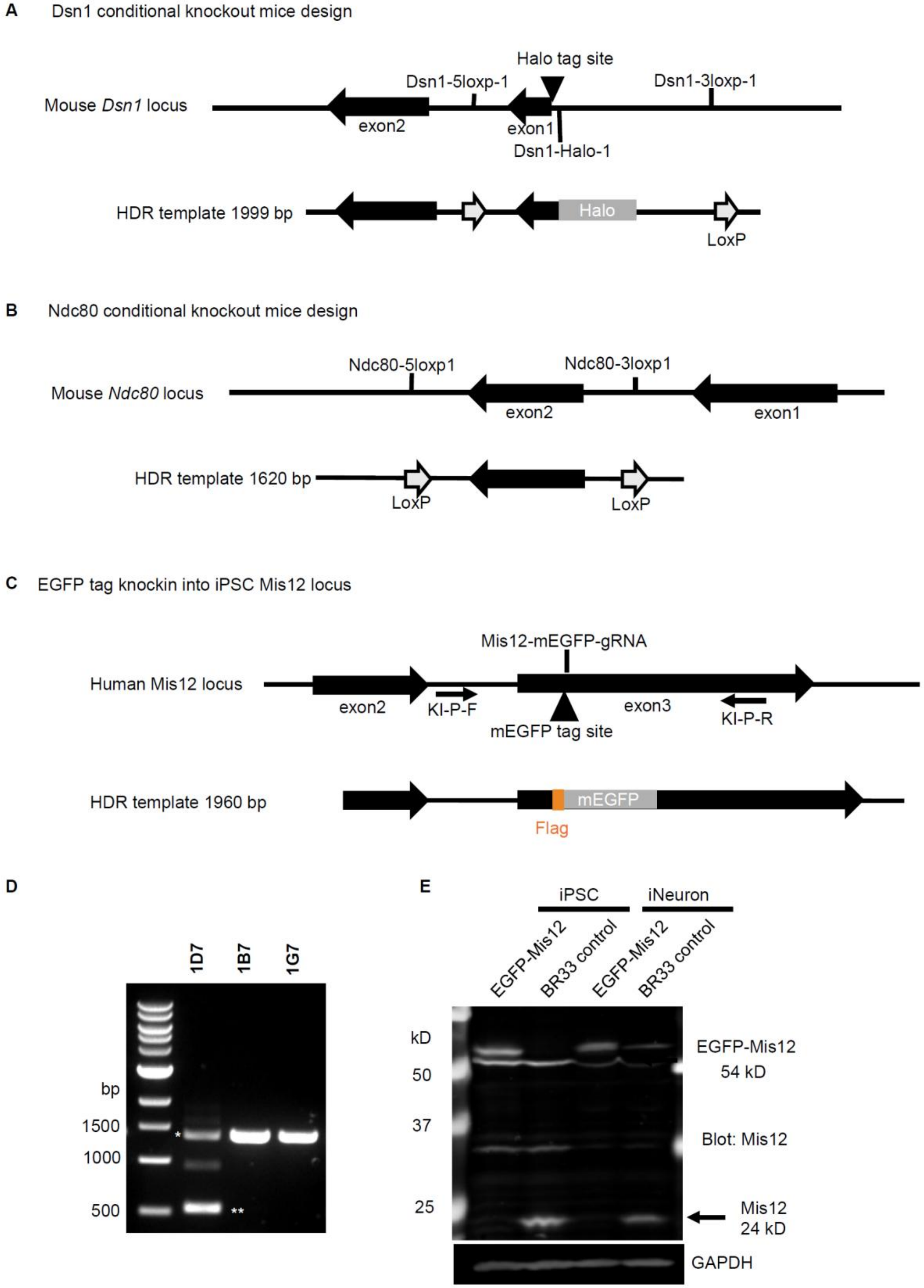

Figure S1. Generation of floxed mice at the Dsn1 and Ndc80 locus.
(A) Illustration of generating the conditional Dsn1 allele. The introns flanking the first exon of Dsn1 were targeted by two gRNAs close to the loxP insertion sites. Another gRNA targeted a site close to the start codon for inserting the Halo tag. A single DNA block was designed to carry the two loxP sequences, Halo tag coding sequence, and two homology arms for directing the CRISPR editing.
(B) Illustration of generating the conditional Ndc80 allele. The introns flanking the second exon of Ndc80 were targeted by two gRNAs close to the loxP insertion sites. A single DNA block was designed to carry the two loxP sequences, and two homology arms that directed the CRISPR editing.
(C) Illustration of generating the EGFP-tagged Mis12 allele in the human BR33 iPSC line. The start codon was targeted by the gRNA and a single DNA block was designed to carry the two homology arms, Flag and EGFP coding sequence for CRISPR editing. The primers used for identification of insertions lines are indicated (KI-P-F and KI-P-R).
(D) PCR verification of the EGFP insertion at the Mis12 locus, using the primers indicated in (C). The amplified band of wildtype is 536 bp (**) and with the GFP insertion is 1373 bp (*). Clone 1D7 is heterozygous and 1B7 and 1G7 are homozygous for the insertion.
(E) MIS12 expression in wildtype and EGFP-tagged iPSCs and iNeurons. Western blot was probed with an antiserum raised against the human MIS12 complex (44). The parental BR33 line contains the expected band of 24kD which is shifted to 54 kDa in the homozygous IB7 EGFP-Mis12 knock-in line. A cross-reacting band at 52 kD is present in both the parental and knock-in iPSCs.

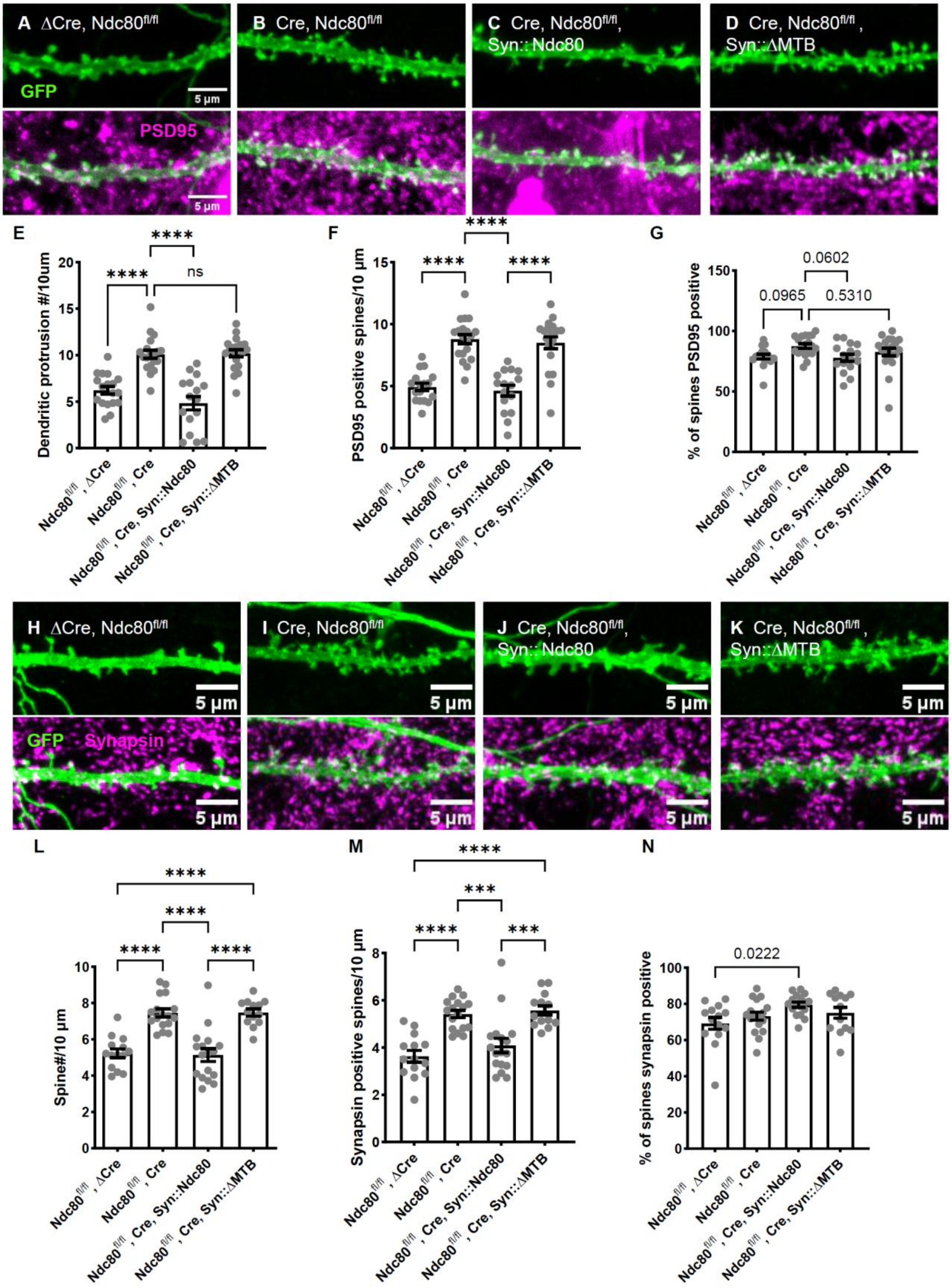
A ΔCre, Ndc80fl/fl
B Cre, Ndc80fl/fl
C Cre, Ndc80fl/fl, Syn:: Ndc80
D Cre, Ndc80fl/fl, Syn::ΔMTB
GFP
PSD95
5 µm
E
F
G
Dendritic protrusion #/10um
PSD95 positive spines/10 µm
% of spines PSD95 positive
****
ns
0.0965
0.0602
0.5310
Ndc80fl/fl, ΔCre
Ndc80fl/fl, Cre
Ndc80fl/fl, Cre, Syn::Ndc80
Ndc80fl/fl, Cre, Syn::ΔMTB
H ΔCre, Ndc80fl/fl
I Cre, Ndc80fl/fl
J Cre, Ndc80fl/fl, Syn:: Ndc80
K Cre, Ndc80fl/fl, Syn::ΔMTB
GFP Synapsin
L
M
N
Spine#/10 µm
Synapsin positive spines/10 µm
% of spines synapsin positive
***
0.0222

Figure S2. Knockout of kinetochore components increased mature dendritic spines in cultured hippocampal neurons.

(A-D) Hippocampal neurons from Ndc80$^{fl/fl}$ mice were infected on DIV19 with lentivirus expressing inactive ∆Cre (A), Cre (B), Cre and a rescue construct expressing wildtype NDC80 (C) or Cre with rescue virus expressing a microtubule-binding deficient NDC80 (∆MTB-NDC80) (D). Neurons were subsequently transfected with a plasmid expressing membrane-bound GFP on DIV21. After fixation on DIV24, neurons were stained with anti-GFP (green) and anti-PSD95 (magenta). (E-G) Dendritic spines (E) and spines with PSD95 puncta were quantified (F) from images as in (A-D) and the percentage of spines positive for PSD95 was calculated for each condition (G). (H-K) Hippocampal neurons from Ndc80$^{fl/fl}$ mice were infected on DIV14 with lentivirus expressing inactive ∆Cre (H), Cre (I), Cre and a rescue construct expressing wildtype NDC80 (J) or microtubule-binding deficient NDC80 (∆MTB-NDC80) (K). Neurons were subsequently transfected with a plasmid expressing membrane-bound GFP on DIV16. After fixation on DIV19, neurons were stained with anti-GFP (green) and anti-synapsin (magenta). (L-N) Dendritic spines (L) and spines with adjacent synapsin puncta (M) were quantified from images as in (H-K) and the percentage of spines with adjacent synapsin puncta was calculated for each condition (N). Data represent mean ± SEM; each dot represents one neuron. One-way ANOVA with Tukey's multiple comparisons test was performed for the quantification and adjusted p values are shown. *** $p < 0.001$ **** $p < 0.0001$.

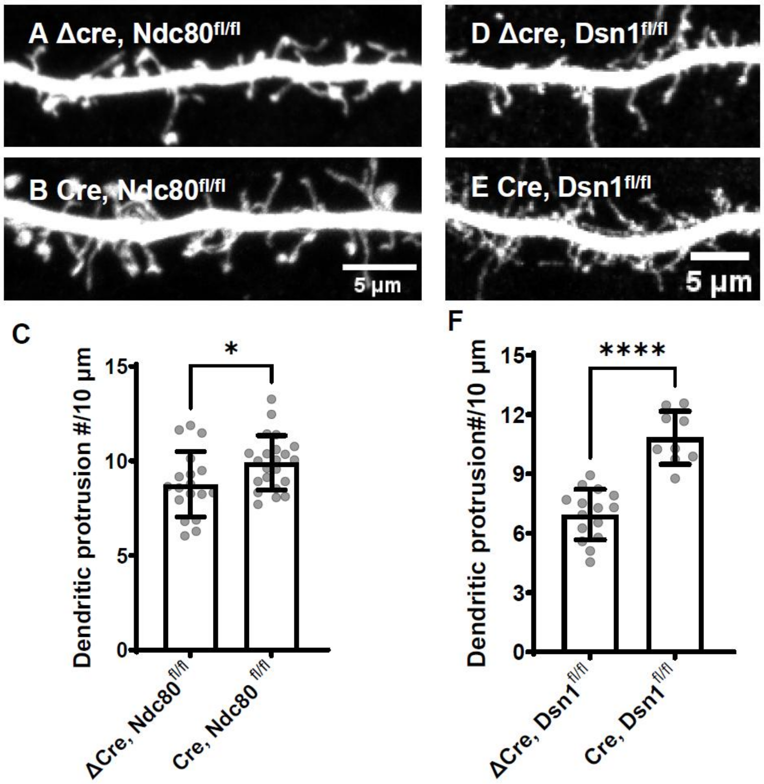

Figure S3. Loss of Ndc80 or Dsn1 increases dendritic spines in cortical neurons.
(A - C) Cortical neurons from $Ndc80^{fl/fl}$ mice were infected on DIV10 with lentivirus expressing inactive ∆Cre (A) or Cre (B) and transfected with plasmid expressing membrane bound GFP on DIV12. Neurons were fixed on DIV14 and dendritic spines were quantified according to the GFP signal (C). (D-F) Cortical neurons from $Dsn1^{fl/fl}$ mice were grown and transfected as above, and dendritic spines were quantified. Data represent mean ± SEM; each dot represents one neuron. Welch's t test was performed for the quantification. **** $p < 0.0001$, * $p < 0.05$.

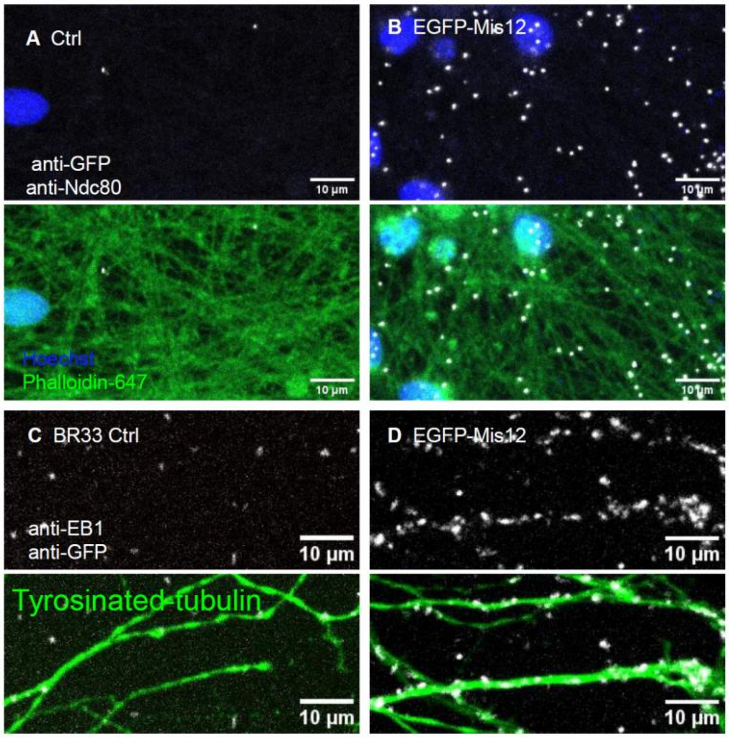

Figure S4. Proximity ligation assay showing colocalization of MIS12 and the NDC80 complex, and MIS12 and EB1 in iNeuron neurites. (A and B) Control BR33 (A) and EGFP-MIS12 knock-in (B) iNeurons labeled with the PLA product (white) formed between anti-NDC80 complex and anti-GFP on D20 (20 days after differentiation). The neurites are visualized with phalloidin-Atta-647N to label F-actin (green) and Hoechst dye to label nuclei (blue). (C and D) Control BR33 (C) and EGFP-MIS12 knock-in (D) iNeurons labeled with the PLA product (white) formed between anti-EB1 and anti-GFP on D11. The neurites are visualized with tyrosinated tubulin (green) and Hoechst dye to label nuclei (blue).

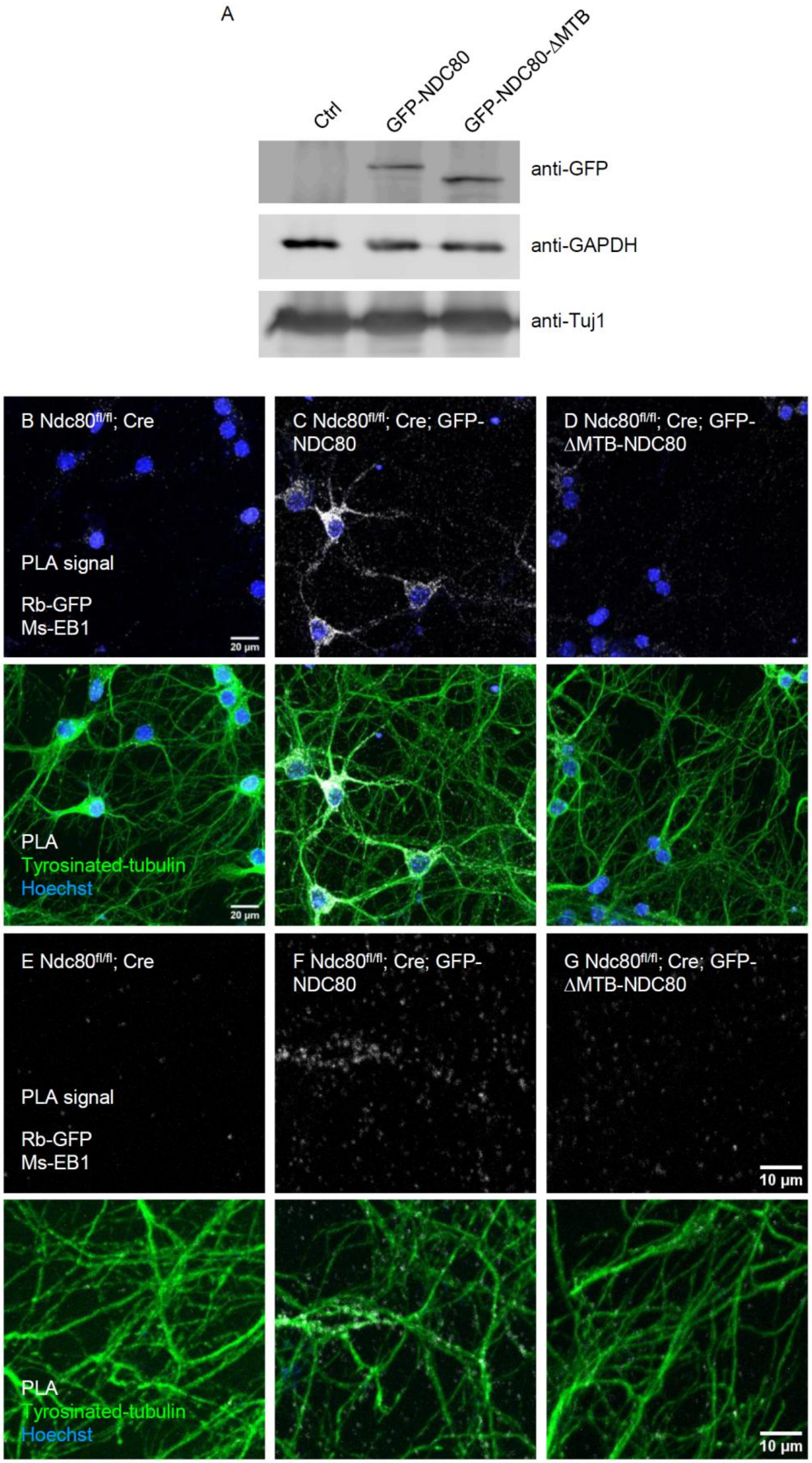
A
Ctrl
GFP-NDC80
GFP-NDC80-ΔMTB
anti-GFP
anti-GAPDH
anti-Tuj1
B Ndc80fl/fl; Cre
C Ndc80fl/fl; Cre; GFP-NDC80
D Ndc80fl/fl; Cre; GFP-ΔMTB-NDC80
PLA signal
Rb-GFP
Ms-EB1
20 µm
PLA
Tyrosinated-tubulin
Hoechst
20 µm
E Ndc80fl/fl; Cre
F Ndc80fl/fl; Cre; GFP-NDC80
G Ndc80fl/fl; Cre; GFP-ΔMTB-NDC80
PLA signal
Rb-GFP
Ms-EB1
10 µm
PLA
Tyrosinated-tubulin
Hoechst
10 µm

Figure S5. Proximity ligation assay showing that the microtubule-binding deficient form of NDC80 localizes less than wildtype NDC80 with EB1 in DIV7 mouse neurons. A. Western blot showing that the GFP-tagged wildtype NDC80 and microtubule binding deficient form NDC80 (-ΔMTB-NDC80) have similar expression in mouse neurons. (B-G) Control (B and E), GFP-NDC80 expressing (C and F), and GFP-ΔMTB-NDC80 expressing (D and G) neurons labeled with the PLA product (white) formed between anti-EB1 and anti-GFP. The neurites are visualized with tyrosinated tubulin (green) and Hoechst dye to label nuclei (blue). (E-G) show higher magnification images of neurites from panels (B-D).

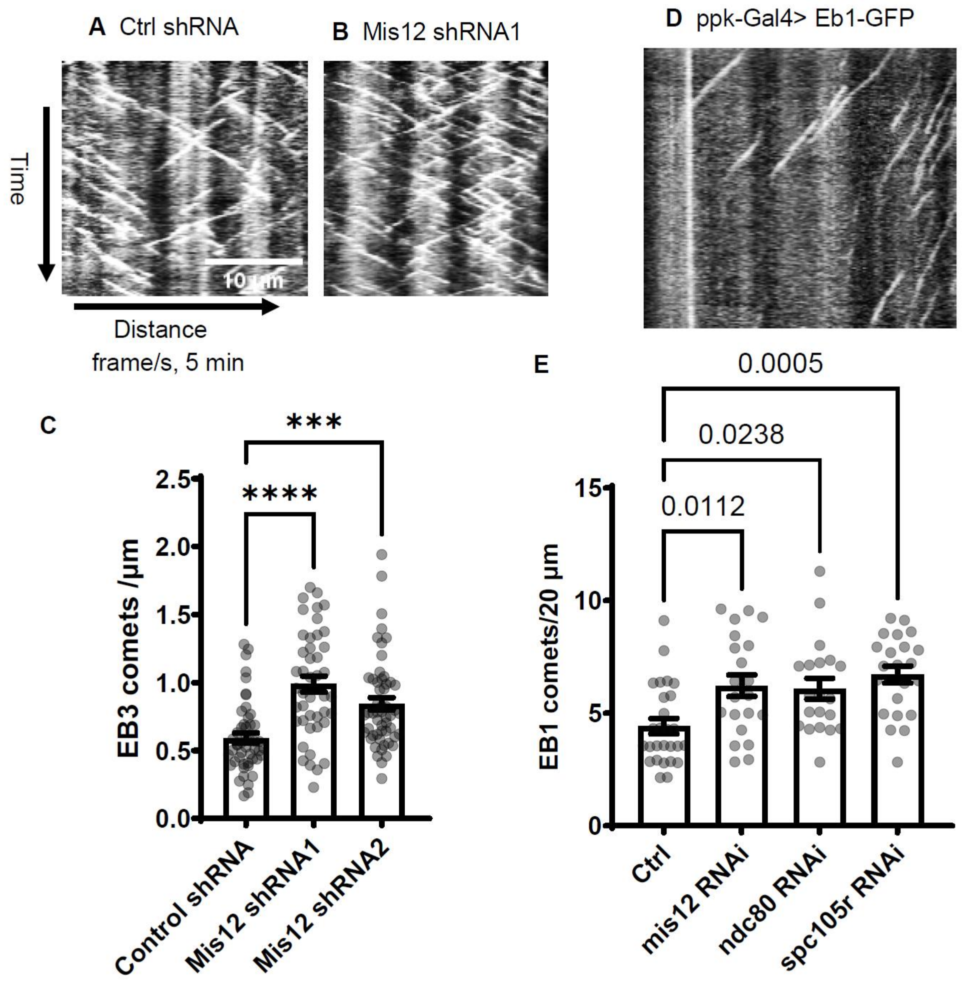

Figure S6. Increased plus-end comets upon knockdown of kinetochore components in rat and Drosophila neurons. (A and B) rat hippocampal neurons transfected by EB3-GFP and either control shRNA (A) or Mis12 shRNA (B) on DIV6. EB3 comets were live-imaged on DIV11 and quantified in (C). (D,E) Kymograph (D and quantification (E) of ppk-Gal4 driven EB1-GFP comets in *Drosophila* third-instar larval sensory neurons. ppk-Gal4 driven RNAi was used to knockdown expression of mis12, Ndc80, or spc105r in these neurons. A representative recording of comets from a control sensory dendrite is shown in (D). Mean ± SEM are indicated; each dot represents one neuron. One-way ANOVA with Tukey's multiple comparisons test was performed and adjusted p value are shown. **** $p < 0.0001$, *** $p < 0.001$.

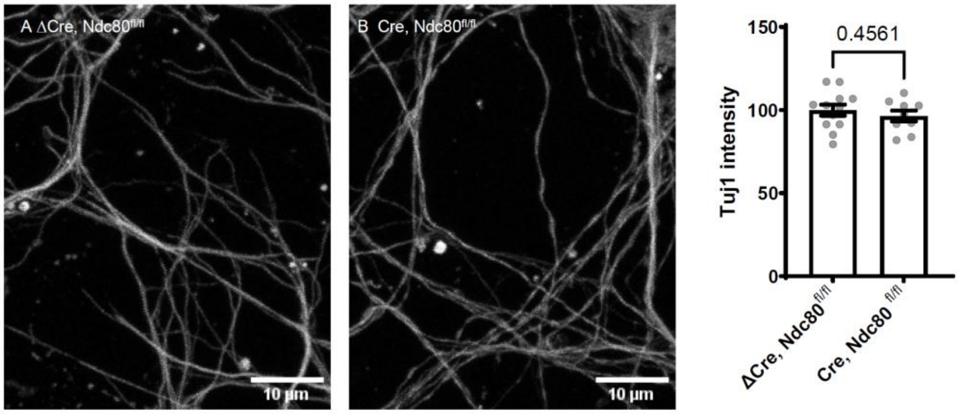

Figure S7. Tubulin immunostaining of hippocampal neurons. Cultures of $Ndc80^{fl/fl}$ hippocampal neurons were infected with lentivirus expressing inactive ∆Cre (A) or Cre (B) as in Figure 5. Cultures were stained with Tuj1 anti-tubulin. Tubulin immunoreactivity was quantified. Mean ± SEM are indicated; each dot represents a single field and indicated p value from Student's T-test.

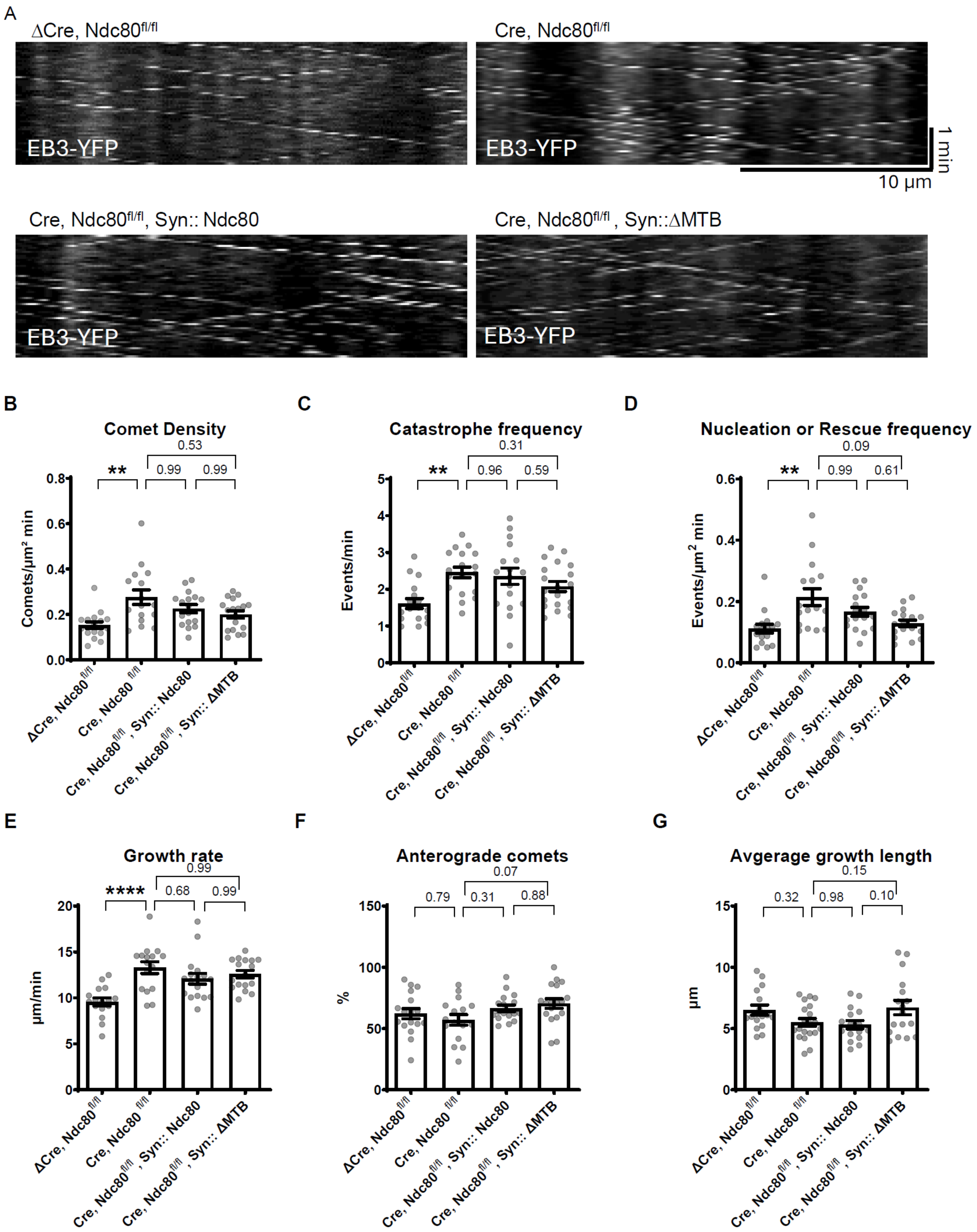
A
ΔCre, Ndc80fl/fl
Cre, Ndc80fl/fl
EB3-YFP
1 min
10 μm
Cre, Ndc80fl/fl, Syn:: Ndc80
Cre, Ndc80fl/fl , Syn::ΔMTB
B
Comet Density
Comets/μm² min
C
Catastrophe frequency
Events/min
D
Nucleation or Rescue frequency
Events/μm² min
E
Growth rate
μm/min
F
Anterograde comets
%
G
Avgerage growth length
μm
ΔCre, Ndc80fl/fl
Cre, Ndc80 fl/fl
Cre, Ndc80fl/fl, Syn:: Ndc80
Cre, Ndc80fl/fl, Syn:: ΔMTB

Figure S8. Ndc80 interaction with microtubules modulates their dynamic behavior.
A) Representative kymographs of EB3-YFP comets at the plus ends of growing microtubules in a dendritic segment of 18 DIV Ndc80$^{flox/flox}$ hippocampal neurons expressing inactive DCre, Cre, Cre + Ndc80 or Cre + Ndc80 ΔMTB. Scale bar: horizontal, 10 μm; vertical, 1 min.
(B - G) Quantification of microtubule-dynamics parameters including comet density (B), catastrophe frequency (C), nucleation or rescue frequency (D), microtubule growth rate (E), percentage of anterograde comets (F), and average growth length (G) of Ndc80$^{flox/flox}$ neurons of each condition, from at least three different neuronal cultures. Kruskal-Wallis test with Dunn's multiple comparison test was used for B, D and E. One Way ANOVA with Tukey's multiple comparisons test was use for C, F and G. Adjusted p values are shown **** $p < 0.0001$, ** $p < 0.01$.

Video S1. Microtubule dynamics in DIV18 Ndc80$^{flox/flox}$ hippocampal neurons expressing DCre. Time-lapse video microscopy of a dendritic segment of a Ndc80$^{fl/fl}$ hippocampal neuron expressing LifeAct-RFP (magenta in the upper panel) and EB3-YFP (green in the upper panel, gray in the lower panel), infected with DCre- encoding lentivirus. Images were collected at 1 frame every 5 s for 5 min. Arrows indicate microtubules invading dendritic spines. Scale bar = 5 μm.
Video S2. Microtubule dynamics in DIV18 Ndc80$^{flox/flox}$ 1 hippocampal neurons expressing Cre. Time-lapse video microscopy of a dendritic segment of a Ndc80$^{fl/fl}$ hippocampal neuron expressing LifeAct-RFP (magenta in the upper panel) and EB3-YFP (green in the upper panel, gray in the lower panel), infected with Cre- encoding lentivirus. Images were collected at 1 frame every 5 s for 5 min. Arrows indicate microtubules invading dendritic spines. Scale bar = 5 μm.
Video S3. Microtubule dynamics in DIV18 Ndc80$^{flox/flox}$ 1 hippocampal neurons expressing Cre and full length Ndc80. Time-lapse video microscopy of a dendritic segment of a Ndc80$^{fl/fl}$ hippocampal neuron expressing LifeAct-RFP (magenta in the upper panel) and EB3-YFP (green in the upper panel, gray in the lower panel) and infected with lentivirus expressing Cre and full-length Ndc80. Images were collected at 1 frame every 5 s for 5 min. Arrows indicate microtubules invading dendritic spines. Scale bar = 5 μm.
Video S4. Microtubule dynamics in DIV18 Ndc80$^{flox/flox}$ 1 hippocampal neurons expressing Cre and Ndc80 lacking the microtubule-binding region. Time-lapse video microscopy of a dendritic segment of a Ndc80$^{fl/fl}$ hippocampal neuron expressing LifeAct-RFP (magenta in the upper panel) and EB3-YFP (green in the upper panel, gray in the lower panel) and infected with lentivirus expressing Cre and Ndc80 ΔMTB. Images were collected at 1 frame every 5 s for 5 min. Arrows indicate microtubules invading dendritic spines. Scale bar = 5 μm.

SI References

1. E. A. Pollina *et al.*, A NPAS4-NuA4 complex couples synaptic activity to DNA repair. *Nature* **614**, 732–741 (2023).
2. T. Aida *et al.*, Cloning-free CRISPR/Cas system facilitates functional cassette knock-in in mice. *Genome biology* **16**, 87 (2015).
3. K. S. Ghanta, T. Ishidate, C. C. Mello, Microinjection for precision genome editing in Caenorhabditis elegans. *STAR Protoc* **2**, 100748 (2021).
4. F. Rago, K. E. Gascoigne, I. M. Cheeseman, Distinct organization and regulation of the outer kinetochore KMN network downstream of CENP-C and CENP-T. *Curr Biol* **25**, 671–677 (2015).
5. Y. Zhang *et al.*, Rapid single-step induction of functional neurons from human pluripotent stem cells. *Neuron* **78**, 785–798 (2013).
6. B. A. Stewart, H. L. Atwood, J. J. Renger, J. Wang, C. F. Wu, Improved stability of Drosophila larval neuromuscular preparations in haemolymph-like physiological solutions. *J Comp Physiol A* **175**, 179–191 (1994).
7. S. F. B. van Beuningen *et al.*, TRIM46 Controls Neuronal Polarity and Axon Specification by Driving the Formation of Parallel Microtubule Arrays. *Neuron* **88**, 1208–1226 (2015).
